# Optimal prediction with resource constraints using the information bottleneck

**DOI:** 10.1371/journal.pcbi.1008743

**Published:** 2021-03-08

**Authors:** Vedant Sachdeva, Thierry Mora, Aleksandra M. Walczak, Stephanie E. Palmer

**Affiliations:** 1 Graduate Program in Biophysical Sciences, University of Chicago, Chicago, Illinois, United States of America; 2 Laboratoire de physique de l’École normale supérieure, Centre National de la Recherche Scientifique, Paris, France; 3 Paris Sciences et Lettres University Paris, Paris, France; 4 Sorbonne Université Paris, Paris, France; 5 Université de Paris, Paris, France; 6 Department of Organismal Biology and Anatomy, University of Chicago, Chicago, Illinois, United States of America; 7 Department of Physics, University of Chicago, Chicago, Illinois, United States of America; Imperial College London, UNITED KINGDOM

## Abstract

Responding to stimuli requires that organisms encode information about the external world. Not all parts of the input are important for behavior, and resource limitations demand that signals be compressed. Prediction of the future input is widely beneficial in many biological systems. We compute the trade-offs between representing the past faithfully and predicting the future using the information bottleneck approach, for input dynamics with different levels of complexity. For motion prediction, we show that, depending on the parameters in the input dynamics, velocity or position information is more useful for accurate prediction. We show which motion representations are easiest to re-use for accurate prediction in other motion contexts, and identify and quantify those with the highest transferability. For non-Markovian dynamics, we explore the role of long-term memory in shaping the internal representation. Lastly, we show that prediction in evolutionary population dynamics is linked to clustering allele frequencies into non-overlapping memories.

## Author summary

From catching a ball to building immunity, we rely on the ability of biological systems to incorporate past observations to make predictions about the future state of the environment. However, the success of these predictions is limited by environmental parameters and encoding capacities of the predictors. We explore these trade-offs in three systems: simple intertial motion, more complex motion with long-tailed temporal correlations, and mutating viral strains. We show that the velocity and position of a moving object should not be equally well-remembered in the biological systems internal representation, and identify the flexible “best-compromise” representations that are not optimal but remain predictable in a wide range of parameters regimes. In the evolutionary context, we find that the optimal predictive representations are discrete, reminiscent of immune strategies that cover the space of potential viruses.

## 1 Introduction

How biological systems represent external stimuli is critical to their behavior. The efficient coding hypothesis, which states that neural systems extract as much information as possible from the external world, given basic encoding capacity constraints, has been successful in explaining some early sensory representations in the brain. Barlow suggested sensory circuits may reduce redundancy in the neural code and minimize metabolic costs for signal transmission [1–4]. However, not all external stimuli are as important to an organism, and behavioral and environmental constraints need to be integrated into this picture to more broadly characterize biological encoding. Delays in signal transduction in biological systems mean that predicting external stimuli efficiently can confer benefits to biological systems [5–8], making prediction a general goal in biological sensing.

Evidence that representations constructed by sensory systems efficiently encode predictive information has been found in the visual and olfactory systems [9–11]. Molecular networks have also been shown to be predictive of future states, suggesting prediction may be one of the fundamental principles of biological computation [12, 13]. However, the coding capacity of biological systems is limited because they cannot provide arbitrarily high precision about their inputs: limited metabolic resources and other sources of internal noise impose finite-precision signal encoding. Given these trade-offs, one way to efficiently encode the history of an external stimulus is to keep only the information relevant for the prediction of the future input [13–15]. Here, we explore how optimal predictions might be encoded by neural and molecular systems using a variety of dynamical inputs that explore a range of temporal correlation structures. We solve the ‘information bottleneck’ problem in each of these scenarios and describe the optimal encoding structure in each case [14].

The information bottleneck framework, introduced by Tishby and colleagues [14, 16–18], allows us to define a ‘relevance’ variable in the encoded sensory stream. We take the relevant piece to be the future behavior of that input. Solving the bottleneck problem allows us to optimally estimate the future state of the external stimulus, given a certain amount of information retained about the past. In general, predicting the future coordinates of a system, *X*_*t*+Δ*t*_ reduces to knowing the precise historical coordinates of the stimulus *X*_*t*_ and an exact knowledge of the temporal correlations in the system. These rules and temporal correlations can be thought of as arising from two parts: a deterministic portion, described by a function of the previous coordinates, $H(X_{t})$, and the noise internal to the system, *ξ*(*t*). Knowing the actual realization of the noise *ξ*(*t*) reduces the prediction problem to simply integrating the stochastic equations of motion forward in time. If the exact realization of the noise if not known, we can still perform a probabalistic prediction by calculating the future form of the probability distribution of the variable *X*_*t*_ or its moments [19, 20]. The higher-order moments yield an estimate of *X*_*t*_ and the uncertainty in the estimate. However, biological systems cannot precisely know *X*_*t*_ due to inherently limited readout precision [21, 22], creating a trade-off between representing the past and predicting the future.

**Fig 1:**
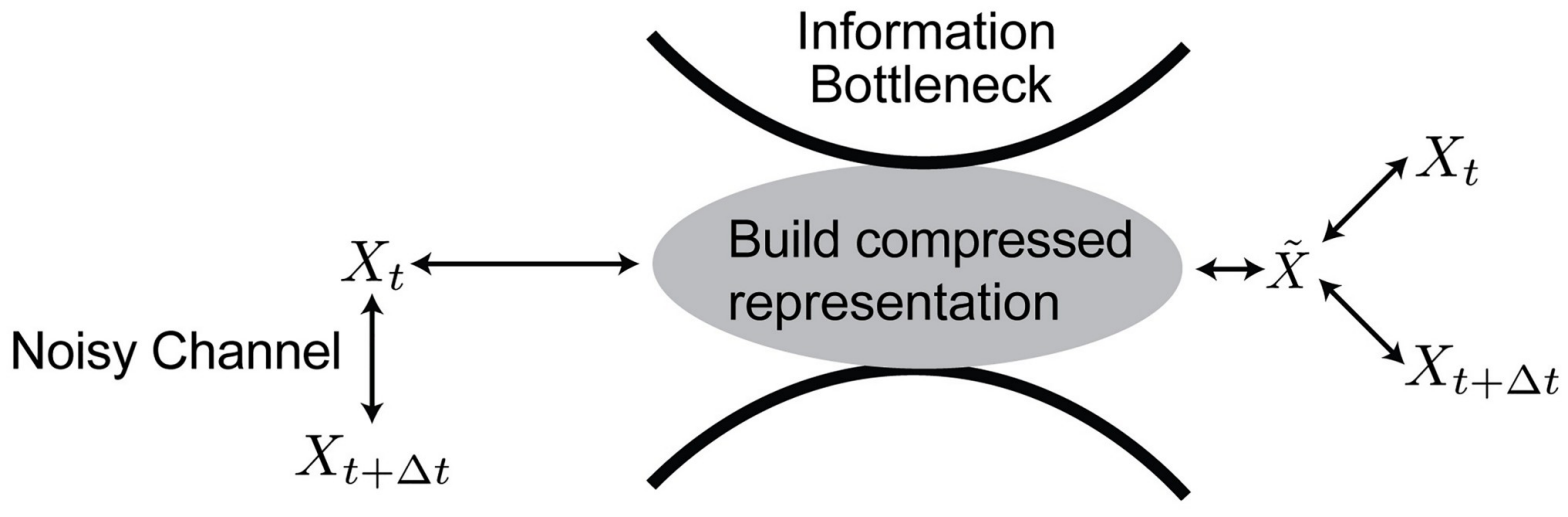
A schematic representation our predictive information bottleneck. On the left hand side, we have coordinates *X*_*t*_ evolving in time, subject to noise to give *X*_*t*+Δ*t*_. We construct a representation, $\tilde{X}$, that compresses the *X*_*t*_ (minimizes $I(X_{t};\tilde{X})$) while retaining as much information about *X*_*t*+Δ*t*_ (maximizes $I(\tilde{X};X_{t+\Delta t})$) up to the weighting of the prediction compared to the compression set by *β*.

We briefly summarize the information bottleneck method to quantify this trade-off here, and provide a more thorough explanation of the case with Gaussian statistics (reproduced from [16]) in S1 Text. The method assumes that the input variable, in our case the signal *X*_*t*−*t*_0_:*t*_, which considers measurements between times *t* − *t*_0_ and *t*. We will call the past. This can be used to make inferences about the relevance variable, in our case the future signal $X_{t+\Delta t:t+\Delta t+t_{0}}$, which considers measurements between times *t* + Δ*t* and *t* + Δ*t* + *t*_0_. We will call this the future. For convenience, in this introduction, we will take the past as a single point in time, *X*_*t*_ and the future as *X*_*t*+Δ*t*_. The resource constraints are introduced via a representation variable, $\tilde{X}$, which can have a varying amount of information about the input signal, *X*_*t*_. This $\tilde{X}$, which has a dependence on the input, $P(\tilde{X}|X_{t})$, is constrained to be maximally informative of the future signal, subject to a constraint on $I(X_{t};\tilde{X})$, the information it has about the past (Fig 1).

Formally, this representation is constructed by optimizing the objective function,

$$\begin{array}{c} \min_{P(\tilde{X}|X_{t})}L\left[P\left(\tilde{X}|X_{t}\right)\right]=I\left(X_{t};\tilde{X}\right)-\beta I\left(\tilde{X};X_{t+\Delta t}\right). \end{array}$$ (1)

Each term is the mutual information between two variables: the first between the *X*_*t*_ and estimate of *X*_*t*_ given our representation model, $\tilde{X}$, and the second between $\tilde{X}$ and future input. The tradeoff parameter, *β*, controls how much future information we want $\tilde{X}$ to retain as it is maximally compressed. For large *β*, $\tilde{X}$ must be maximally informative about *X*_*t*+Δ*t*_, and will have, in general, the lowest compression. Small *β* means less information is retained about the future and high, lossy compression is allowed.

The causal relationship between *X*_*t*_ and *X*_*t*+Δ*t*_ results in a data processing inequality, $I\left(X_{t};\tilde{X}\right)\geq I\left(X_{t+\Delta t};\tilde{X}\right)$, meaning that the information generated about *X*_*t*+Δ*t*_ cannot exceed the amount encoded about *X*_*t*_ [23]. Additionally, the information about *X*_*t*_ that the representation can extract is bounded by the amount of information *X*_*t*_, itself, contains about the *X*_*t*+Δ*t*_, $I\left(\tilde{X};X_{t+\Delta t}\right)\leq I\left(X_{t};X_{t+\Delta t}\right)$.

We use this framework to study how biological systems can optimally encode external stimuli for downstream decoding, but without any explicit constraints on or specification of that decoder. Here, we assume that the compressed representation variable has a one-time-step output and only has access to a fixed amount of historical information about the stimulus. Here, we assume that the compressed representation variable has a single ‘present’ time-step output and only has access to a fixed amount of historical information about the stimulus. This reflects, for example, the instantaneous neural output from a retinal ganglion cell population that is passed downstream to the cortex for further processing and readout. We start with a one-time-step past input and then extend this to a longer temporal window into the past. We begin by assuming a one-time-step past input and then later extend it to a more extended temporal window in the past. The optimal predictive encoder does in general favor some aspects of this past information (position information) over others (velocity information). A downstream decoder may be able to recover some of the lower priority information by combining measurements and predictions across time to reduce variance post hoc, but the gain in precision comes at the cost of additional constraints on the size and complexity of the encoded representation variable. In addition, the gained information about the stimulus that was originally discarded may not provide significant predictive advantages. We do, however, provide a comparison between our information bottleneck framework and the results of a model that performs this kind of prediction combined with measurement and error estimates across time in Section D in S2 Text. There we demonstrate that for a given level of $I(X_{t};\tilde{X})$, a Kalman filter achieves lower $I(\tilde{X};X_{t+\Delta t})$. A question we do not explore here is how to, practically, read out the optimally encoded representation. It has been shown previously that simple perceptrons can read out predictive information from the retinal code [24], which makes biologically plausible readout possible and is a direction of future work.

We use information bottleneck to compute the optimal predictive encoding in two well-studied dynamical systems with ties to biological data: the stochastically-driven damped harmonic oscillator (SDDHO) and the Wright-Fisher model. We look at these two different systems to gain intuition about how different types of dynamics influence the ability of a finite and noisy system to make accurate predictions. We further consider two types of SDDHO processes to study the effects of noise correlations on prediction. Our exploration of the SDDHO system has a two-fold motivation: it is a physical system that describes motion that a visual system might need to process and predict to catch prey or evade predators. It is also the simplest possible continuous stochastic system whose full dynamics can be solved exactly. Previous studies used the SDDHO process to create moving bar stimuli and quantify retinal prediction [10, 24, 25]. Prediction of a time series with Markovian dynamics is not limited to physical motion, of course. The Wright-Fisher model [26] is a canonical model of evolution [27] which has been used to consider how the adaptive immune system predicts the future state of the pathogenic environment [12, 28]. Resource constraints also create trade-offs between representation precision and prediction in the immune system, and finding the general principles that connect prediction in these two contexts can reveal common principles across biological systems and scales.

The results of these information bottleneck calculations in these different dynamical contexts will reveal the form and content of optimally predictive features. These features are matched both to the input parameters and to the level of resource constraints that compress the input. Our results form expectations about what to find in biological systems when the internal representation can be measured (e.g. as in [10]), and the input statistics match the kinds of dynamics studied here. While our results will show what types of feature extraction are expected in systems predicting their inputs optimally, not all systems may be optimized for a broad range of input dynamics. In fact, we assume that natural selection favors encodings that confer just enough predictive capacity to support the organism’s behavioral repertoire. That might mean flexibly predicting in many different environments either over an individual or group migratory lifespan. To help quantify the ‘transferability’ of any optimally predictive encoding scheme, we will develop a metric, *Q*, that tracks how well one representation performs under other input dynamics, where it might not be the absolute optimal, but still performs well. Of course, we only expect our maximally predictive encodings to match biological filters when the system has an intrinsic behavioral goal that requires prediction. There are computations that do not require prediction, and would presumably result from constraints that prioritize other types of information in the input.

## 2 Results

### 2.1 The stochastically driven damped harmonic oscillator

Previous work explored the ability of the retina to construct an optimally predictive internal representation of a dynamic stimulus. Palmer et al [10] recorded the response of a salamander retina to a moving bar stimulus with SDDHO dynamics. In this case, the spike trains in the retina encode information about the past stimuli in a near-optimally predictive way [10]. In order for optimal prediction to be possible, the retina should encode the position and velocity as dictated by the information bottleneck solution to the problem, for the retina’s given level of compression of the visual input. In that study, the SDDHO was set near critical damping, and only one set of parameters in the model was shown to the retina. Inspired by this experiment, we explore the optimal predictive encoding schemes as a function of the parameters in the dynamics, and we describe the optimal solution across the entire parameter space of the model, over a wide range of desired prediction timescales.

**Fig 2:**
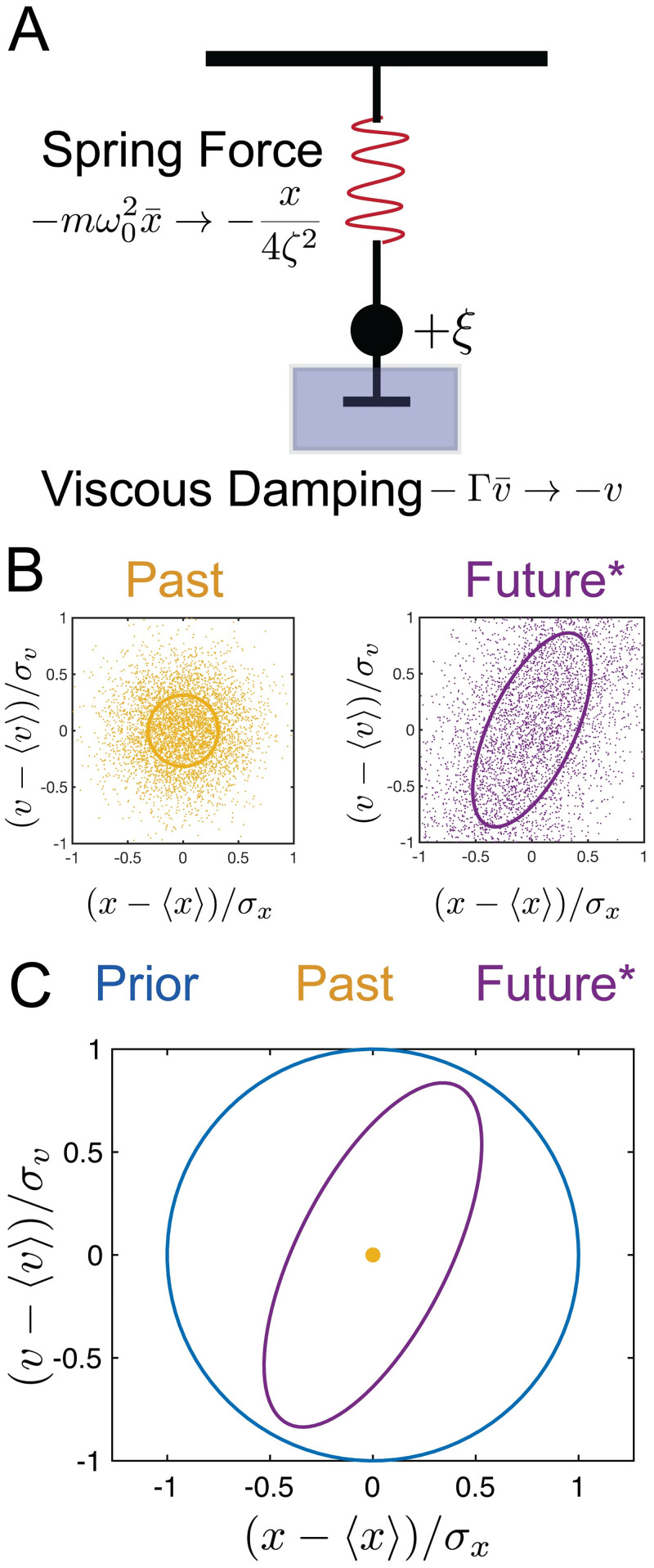
Schematic of the stochastically driven damped harmonic oscillator (SDDHO). (a) The SDDHO consists of a mass attached to a spring undergoing viscous damping and experiencing Gaussian thermal noise of magnitude. There are two parameters to be explored in this model: $\zeta =\frac{1}{2\omega_{0}\tau }$ and $\Delta t=\frac{\Delta t}{\tau }$. (b) $\zeta =\frac{1}{2}$, Δ*t* = 1. Here, we show an example distribution of the history (yellow, left) and show its time evolution (purple, right). We take 5000 samples from the distribution, at random, and let these points evolve in time according to the SDDHO equation of motion. We visualize the evolution of the distribution of points in time via an ellipse representing the 1 − Σ confidence region of the rescaled position and velocity. (c) We illustrate the limiting case of the information bottleneck method when *β* → ∞. Representations of the past and how that constrains an estimate of the future position and velocity of the object can be compared to the prior be examining the relative size and shape of their respective ellipses. The blue circle represents the prior and its 1 − Σ confidence region. In yellow, we plot the inferred 1 − Σ confidence interval associated with the estimate of past, *X*_*t*_, given by the encoding distribution when *β* → ∞. In this limit, the distribution is reduced to a single point. In purple, we plot the 1 − Σ confidence region of *X*_*t*+Δ*t*_ given our knowledge of *X*_*t*_. Precise knowledge of the past coordinates reduces the our uncertainty about the future position and velocity (as compared to the prior), as depicted by the smaller area of the purple ellipse.

We consider the dynamics of a mass *m* in a viscous medium attached to a spring receiving noisy velocity kicks generated by a temporally uncorrelated Gaussian process, as depicted in Fig 2A. The dynamics of this model were solved previously [29]. See Section A in S2 Text for details. Equations of motion are introduced in terms of physical variables $\bar{x}$, $\bar{v}$, and $\bar{t}$ (bars will be dropped later when referring to rescaled variables), which evolve according to

$$\begin{array}{cc} m\frac{d\bar{v}}{d\bar{t}}= & -k\bar{x}-\Gamma \bar{v}+{\left(2k_{B}T\Gamma \right)}^{1/2}\xi \left(\bar{t}\right),\\ \frac{d\bar{x}}{d\bar{t}}= & \bar{v}, \end{array}$$ (2)

where *k* is the spring constant, Γ the damping parameter, *k*_*B*_ the Boltzmann constant, *T* temperature, $\langle \xi (\bar{t})\rangle =0$, and $\langle \xi \left(\bar{t}\right)\xi \left({\bar{t}}^{\prime }\right)\rangle =\delta \left(\bar{t}-{\bar{t}}^{\prime }\right)$. We rewrite the equation with $\omega_{0}=\sqrt{\frac{k}{m}}$, $\tau =\frac{m}{\Gamma }$, and $D=\frac{k_{B}T}{\Gamma }$. We also introduce a dimensionless parameter, the damping coefficient, *ζ* = 1/(2*ω*_0_ *τ*). When *ζ* < 1, the system is underdamped and the motion of the mass will be oscillatory. When *ζ* ≥ 1, the system is overdamped and the motion will be non-oscillatory. Additionally, the equipartition theorem tells us that $\langle \bar{x}{\left(\bar{t}\right)}^{2}\rangle \equiv x_{0}^{2}=k_{B}T/k=D/\left(\tau \omega_{0}^{2}\right)$. Putting this all together, we obtain

$$\begin{array}{c} \frac{d\bar{v}}{d\bar{t}}=-\frac{\bar{x}}{4\tau^{2}\zeta^{2}}-\frac{\bar{v}}{\tau }+\frac{x_{0}}{\sqrt{2\tau^{3}}\zeta }\xi \left(\bar{t}\right) \end{array}$$ (3)

We make two changes of variable to further simplify our expressions. We set $t=\frac{\bar{t}}{\tau }$ and $x=\frac{\bar{x}}{x_{0}}$. We also define a rescaled velocity, $\frac{dx}{dt}=v$, so that our equation of motion now reads

$$\begin{array}{c} \frac{dv}{dt}=-\frac{x}{4\zeta^{2}}-v+\frac{\xi (t)}{\sqrt{2}\zeta }. \end{array}$$ (4)

There are now just two parameters that govern a particular solution to our information bottleneck problem: *ζ* and Δ*t*, the timescale on which we want to retain optimal information about the future. We define *X*_*t*_ = (*x*(*t*), *v*(*t*)) and *X*_*t*+Δ*t*_ = (*x*(*t* + Δ*t*), *v*(*t* + Δ*t*)) and seek a representation, $\tilde{X}\left(\zeta ,\Delta t\right)$, that can provide a maximum amount of information about *X*_*t*+Δ*t*_ for a fixed amount of information about *X*_*t*_. By considering position and velocity, our system is Markovian, so extended temporal windows provide no additional information. If we were to ignore velocity in this model, estimates of the future would become suboptimal to the information bottleneck bound. We explore models where extended temporal windows are relevant in Section 2.2. To construct the information bottleneck solution in the case with Gaussian variables, we follow the construction given in [16]. We note that due to the Gaussian structure of the joint distribution of *X*_*t*_ and *X*_*t*+Δ*t*_ for the SDDHO, the problem can be solved analytically. The optimal compressed representation is a noisy linear transform of *X*_*t*_ (see S1 Text) [16],

$$\begin{array}{c} \tilde{X}=A_{\beta }X_{t}+\xi . \end{array}$$ (5)

*A*_*β*_ is a matrix whose elements are a function of *β*, the tradeoff parameter in the information bottleneck objective function, and the statistics of the input and output variables. The added noise term, *ξ*, has the same dimensions as *X*_*t*_ and is a Gaussian variable with zero mean and unit variance.

We calculate the optimal compression, $\tilde{X}$, and its predictive information (see Section B in S2 Text). The coordinates at time *t* and time *t* + Δ*t* in the SDDHO bottleneck problem are jointly Gaussian, which means that the optimal compression can be fully described by its first and second-order statistics. We generalize analytically the results that were numerically obtained in Ref. [10] and explore the full parameter space of this dynamical model and examine all predictive bottleneck solutions, including different desired prediction timescales.

We quantify the efficiency of the representation $\tilde{X}$ in terms of the variance of the following four probability distributions: the prior distribution, $P(X_{t})$, the distribution of *X*_*t*_ conditioned on the compression, $P(X_{t}|\tilde{X})$, the distribution of *X*_*t*+Δ*t*_ conditioned on the compressed variable $P(X_{t+\Delta t}|\tilde{X})$, and the distribution of *X*_*t*+Δ*t*_ conditioned on *X*_*t*_ $P(X_{t+\Delta t}|X_{t})$. We represent the uncertainty reduction, or the mutual information between these two variables, using two dimensional contour plots that depict the variances of the distributions in the ((*x* − 〈*x*〉)/*σ*_*x*_, (*v* − 〈*v*〉)/*σ*_*v*_) plane, where *σ*_*x*_ and *σ*_*v*_ are the standard deviations of the signal distribution $P(X_{t})$. We present example distributions of $P(X_{t}|\tilde{X})$ and $P(X_{t+\Delta t}|\tilde{X})$ in Fig 2B (left, right, respectively).

The representation, $\tilde{X}$, will be at most two-dimensional, with each of its components corresponding to linear combinations of position and velocity. It may be lower dimensional for certain values of *β*. The smallest critical *β* for which the representation remains two-dimensional is given in terms of the smallest eigenvalue of the matrix $\Sigma_{X_{t}|X_{t+\Delta t}}\Sigma_{X_{t}}^{-1}$ as *β*_*c*_ = 1/(1 − min{λ_1_, λ_2_}) (see Section B in S2 Text). $\Sigma_{X_{t}|X_{t+\Delta t}}$ is the covariance matrix of the probability distribution of $P(X_{t}|X_{t+\Delta t})$ and $\Sigma_{X_{t}}$ is the input variance. Below this critical *β*, the compressed representation is one dimensional, $\tilde{X}=k_{1}x+k_{2}v+\text{noise}$, but it is still a combination of position and velocity.

Limiting cases along the information bottleneck curve help build intuition about the optimal compression. If $\tilde{X}$ provides no information about the stimulus (e.g. *β* = 0), the variances of both of the conditional distributions match that of the prior distribution, $P(X_{t})$, which is depicted as a circle of radius 1 (blue circle in Fig 2C). However, if the encoding contains information about *X*_*t*_, the variance of $P(X_{t}|\tilde{X})$ will be reduced compared to the prior. The maximal amount of predictive information, which is reached when *β* → ∞, can be visualized by examining the variance of $P(X_{t+\Delta t}|X_{t})$ (e.g. the purple contour in Fig 2C), which quantifies the correlations in *X*, itself, with no compression. Regardless of how precisely the current state of the stimulus is measured, the uncertainty about the future stimulus cannot be reduced below this minimal variance, because of the noise in the equation of motion.

**Fig 3:**
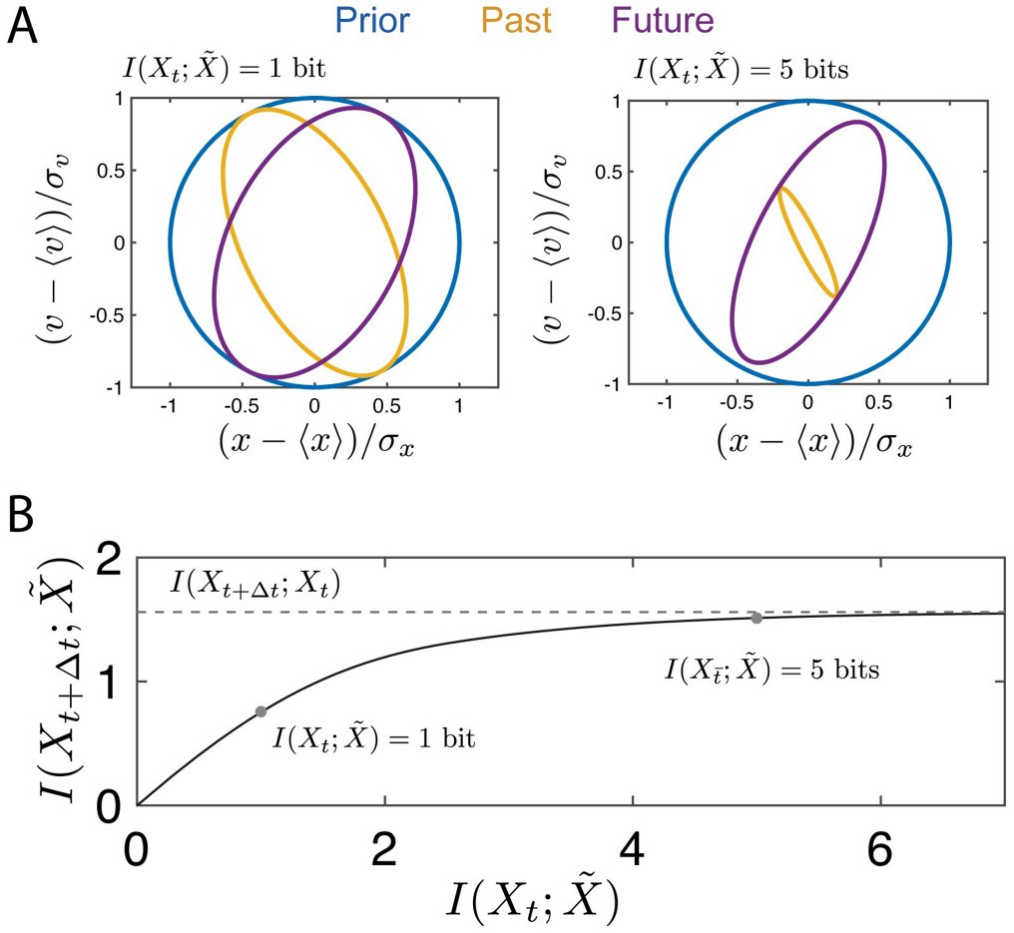
We consider the task of predicting the path of an SDDHO with $\zeta =\frac{1}{2}$ and Δ*t* = 1. (a) (left) We encode the history of the stimulus, *X*_*t*_, with a representation generated by the information bottleneck, $\tilde{X}$, that can store 1 bit of information. Knowledge of the coordinates in the compressed representation space enables us reduce our uncertainty about the bar’s position and velocity, with a confidence interval given by ellipse in yellow. This particular choice of encoding scheme enables us to predict the future, *X*_*t*+Δ*t*_ with a confidence interval given by the purple ellipse. The information bottleneck guarantees this uncertainty in future prediction is minimal for a given level of encoding. (right) The uncertainty in the prediction of the future can be reduced by reducing the overall level of uncertainty in the encoding of the history, as demonstrated by increasing the amount of information $\tilde{X}$ can store about *X*_*t*_. However, the uncertainty in the future prediction cannot be reduced below the variance of the propagator function. (b) We show how the information with *X*_*t*+Δ*t*_scales with the information about *X*_*t*_, highlighting the points represented in panel A.

From Fig 2B, we see that the conditional distribution $P(X_{t+\Delta t}|X_{t})$ is strongly compressed in the position coordinate with some compression in the velocity coordinate. The information bottleneck solution at a fixed compression level (e.g. $I(X_{t};\tilde{X})=1$), shown in Fig 3A (left), gives an optimal encoding strategy for prediction (yellow curve) that reduces uncertainty in the position variable. This yields as much predictive information, $I(X_{t+\Delta t};\tilde{X})$, as possible for this value of $I(X_{t};\tilde{X})$. The uncertainty of the prediction is illustrated by the purple curve. We can explore the full range of compression levels, tracing out an information bottleneck curve for this damping coefficient and desired prediction timescale, as shown in Fig 3. Velocity uncertainty in the compressed representation is only reduced (i.e. predictive information that uses past velocity estimates is only useful) as we allow for less compression, as shown in Fig 3A (right). For both of the cases represented in Fig 3A, the illustrated encoding strategy yields a maximal amount of mutual information between the compressed representation, $\tilde{X}$, and the future for the given level of compression, as indicated by the red dots in Fig 3B.

As noted above, there is a phase transition along the information bottleneck curve, where the optimal, predictive compression of *X*_*t*_ changes from a one-dimensional representation to a two-dimensional one. This phase transition can be pinpointed in *β* for each choice of *ζ* and Δ*t*, and can be determined using the procedure described in is given in the S1 Text. To understand which directions are most important to represent at high levels of compression, we derive the analytic form of the leading eigenvector, *w*_1_, of the matrix $\Sigma_{X_{t}|X_{t+\Delta t}}\Sigma_{X_{t}}^{-1}$. We have defined $\omega^{2}=\frac{1}{4\zeta^{2}}-\frac{1}{4}$ such that

$$\begin{array}{c} w_{1}=[\begin{array}{c} \omega \cot\left(\omega \Delta t\right)+\frac{\left|\csc(\omega \Delta t)\right|}{2\sqrt{2}\zeta }\sqrt{2-\zeta^{2}-\zeta^{2}\cos\left(2\omega \Delta t\right)}\\ 1 \end{array}]. \end{array}$$ (6)

The angle of the encoding vector from the position direction is then given by

$$\begin{array}{c} \phi =\arctan((\omega \cot\left(\omega \Delta t\right)+{\frac{\left|\csc(\omega \Delta t)\right|}{2\sqrt{2}\zeta }\sqrt{2-\zeta^{2}-\zeta^{2}\cos\left(2\omega \Delta t\right)})}^{-1}). \end{array}$$ (7)

We consider *ϕ* in three limits: (I) the small Δ*t* limit, (II) the strongly overdamped limit (*ζ* → ∞), and (III) the strongly underdamped limit (*ζ* → 0).

(I): When *ω*Δ*t* ≪ 1, the angle can be expressed as

$$\begin{array}{c} \phi =\arctan(\frac{\Delta t}{1+\omega^{2}}). \end{array}$$ (8)

This suggests that for small *ω*Δ*t*, the optimal encoding scheme favors position information over velocity information. The change in angle of the orientation from the position axis in this limit goes as *O*(Δ*t*).

(II): The strongly overdamped limit. In this limit, *ϕ* becomes

$$\begin{array}{c} \phi =\arctan(\frac{2\sinh(\frac{\Delta t}{2})}{\cosh\left(\frac{\Delta t}{2}\right)+\sqrt{\frac{1+\cosh(\Delta t)}{2}}}). \end{array}$$ (9)

In the large Δ*t* limit, $\phi \to \frac{\pi }{4}$. In the small Δ*t* limit, *ϕ* → arctan(Δ*t*). Position information is the best predictor of the future input at short lags, which velocity and position require equally fine representation for prediction at longer lags.

(III) The strongly underdamped limit. In this limit, *ϕ* can be written as

$$\begin{array}{c} \phi =\arctan(\frac{2\zeta \sin(\frac{\Delta t}{2\zeta })}{\cos\left(\frac{\Delta t}{2\zeta }\right)+\sqrt{2-\zeta^{2}-\zeta^{2}\cos\left(\frac{\Delta t}{\zeta }\right)}}). \end{array}$$ (10)

We observe periodicity in the optimal encoding angle between position and velocity. This means that the optimal tradeoff between representing position or velocity depends on the timescale of prediction. However, the denominator never approaches 0, so the encoding scheme never favors pure velocity encoding. It returns to position-only encoding when Δ*t*/2*ζ* = *nπ*.

At large compression values, i.e. small amounts of information about *X*_*t*_, the information bottleneck curve is approximately linear. The slope of the information bottleneck curve at small $I(X_{t};\tilde{X})$ is given by 1 − λ_1_, where λ_1_ is the smallest eigenvalue of the matrix, $\Sigma_{X_{t}|X_{t+\Delta t}}\Sigma_{X_{t}}^{-1}$. The value of the slope is

$$\begin{array}{c} 1-\lambda_{1}=\exp\left(-\Delta t\right)\left(\frac{1}{4\omega^{2}\zeta^{2}}+\frac{\cos(2\omega \Delta t)}{4\omega^{2}}+\frac{\left|\sin(\omega \Delta t)\right|}{2\sqrt{2}\omega^{2}\zeta }\sqrt{2-\zeta^{2}-\zeta^{2}\cos\left(2\omega \Delta t\right)}\right). \end{array}$$ (11)

For large Δ*t*, it is clear that the slope will be constrained by the exponential term, and the information will fall as exp(−Δ*t*) as we attempt to predict farther into the future. For small Δ*t*, however, we see that the slope goes as 1 − Δ*t*^2^, and our predictive information decays more slowly.

For vanishingly small compression, i.e. *β* → ∞, the predictive information that can be extracted by $\tilde{X}$ approaches the limit set by the temporal correlations in *X*, itself, given by

$$\begin{array}{c} I\left(X_{t};X_{t+\Delta t}\right)=\frac{1}{2}\log(|\Sigma_{X_{t}}\left|\right)-\frac{1}{2}\log(|\Sigma_{X_{t}|X_{t+\Delta t}}\left|\right). \end{array}$$ (12)

For large Δ*t*, this expression becomes

$$\begin{array}{c} I\left(X_{t};X_{t+\Delta t}\right)\propto \exp\left(-\Delta t\right). \end{array}$$ (13)

For small Δ*t*,

$$\begin{array}{c} I\left(X_{t};X_{t+\Delta t}\right)\propto \Delta t-\frac{1}{2}\log\left(\Delta t\right). \end{array}$$ (14)

The constant term emerges from the physical parameters of the input dynamics.

#### 2.2.1 Optimal representations in all parameter regimes for fixed $I(X_{t};\tilde{X})$

**Fig 4:**
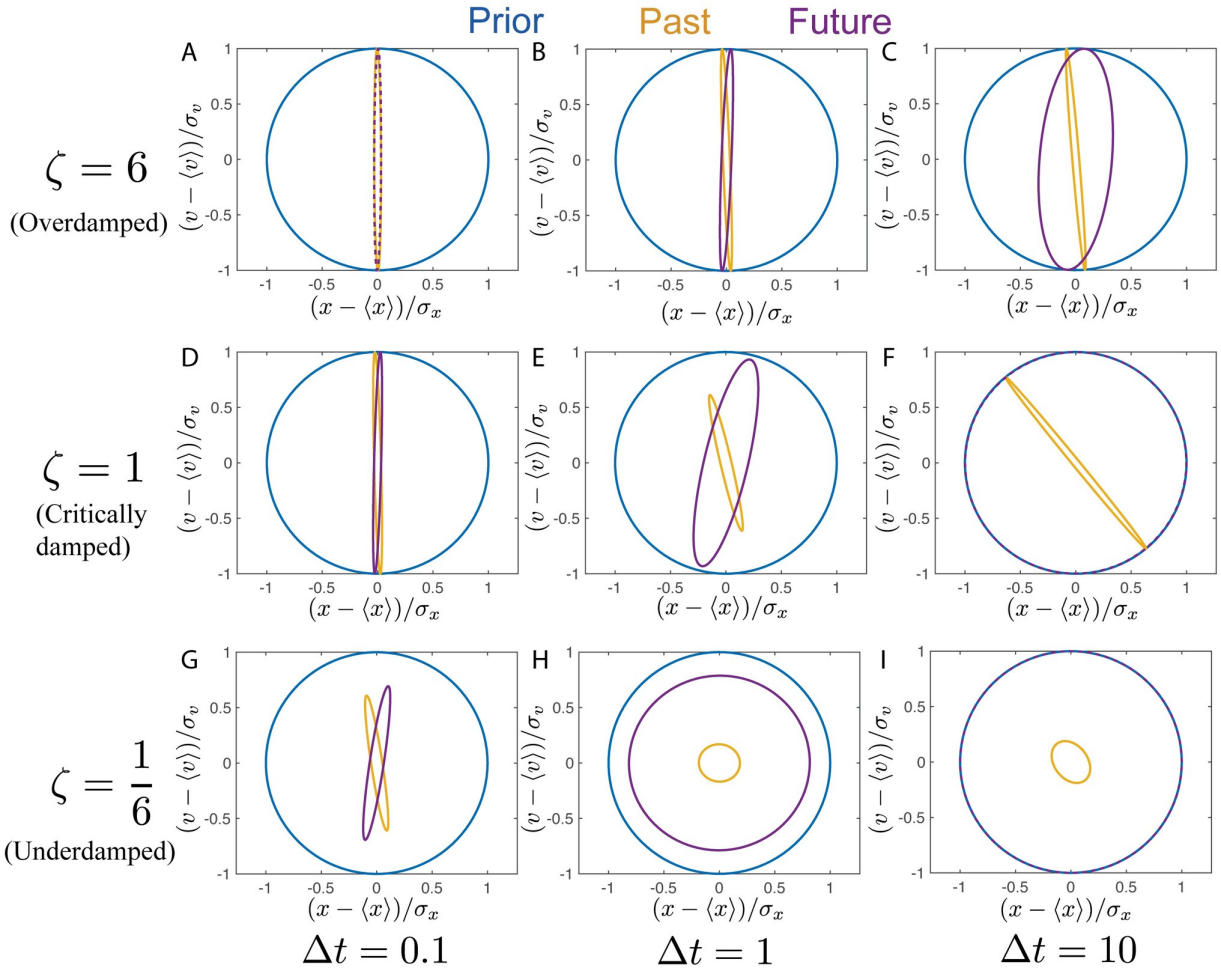
Possible behaviors associated for the SDDHO for a variety of timescales with a fixed $I(X_{t};\tilde{X})$ of 5 bits. For an overdamped SDDHO, panel a-c, the optimal representation continues to encode mostly position information, as velocity is hard to predict. For the underdamped case, panels g-i, as the timescale of prediction increases, the optimal representation changes from being mostly position information to being a mix of position and velocity information. Optimal representations for critically damped input motion are shown in panels d-f. Comparatively, overdamped stimuli do not require precise velocity measurements, even at long timescales. Optimal predictive representations of overdamped input dynamics have higher amounts of predictive information for longer timescales, when compared to underdamped and critically damped cases.

We sweep over all possible parameter regimes of the SDDHO keeping $I(X_{t};\tilde{X})$ fixed at 5 bits and find the optimal representation for a variety of timescales (Fig 4), keeping a fixed amount of information encoded about *X*_*t*_ for each realization of the stimulus and prediction. More information can be transmitted for shorter delays (Fig 4A, 4D and 4G) between the *X*_*t*_ and *X*_*t*+Δ*t*_ signal than for longer delays (Fig 4C, 4F and 4I). In addition, at shorter prediction timescales more information about *X*_*t*_ is needed to reach the upper bound, as more information can be gleaned about the future. In particular, for an overdamped SDDHO at short timescales (Fig 4A), the evolution of the equations of motion are well approximated by integrating Eq 3 with the left hand side set to zero, and the optimal representation encodes mostly position information. This can be visualized by noting that the encoding ellipse remains on-axis and mostly compressed along the position dimension. For the underdamped case, in short time predictions (Fig 4G), a similar strategy is effective. However, for longer predictions (Fig 4H and 4I), inertial effects cause position at one time to be strongly predictive of future velocity and vice versa. As a result, the encoding distribution has to take advantage of these correlations to be optimally predictive. These effects can be observed in the rotation of the encoding ellipse, as it indicates that the uncertainty in position-velocity correlated directions are being reduced, at some cost to position and velocity encoding. The critically damped SDDHO (Fig 4D–4F) demonstrates rapid loss of information about the future, like that observed in the underdamped case. The critically damped case displays a bias towards encoding position over velocity information at both long and intermediate timescales, as in the overdamped case. At long timescales, Fig 4F, the optimal encoding is non-predictive.

#### 2.1.2 Suboptimal representations

**Fig 5:**
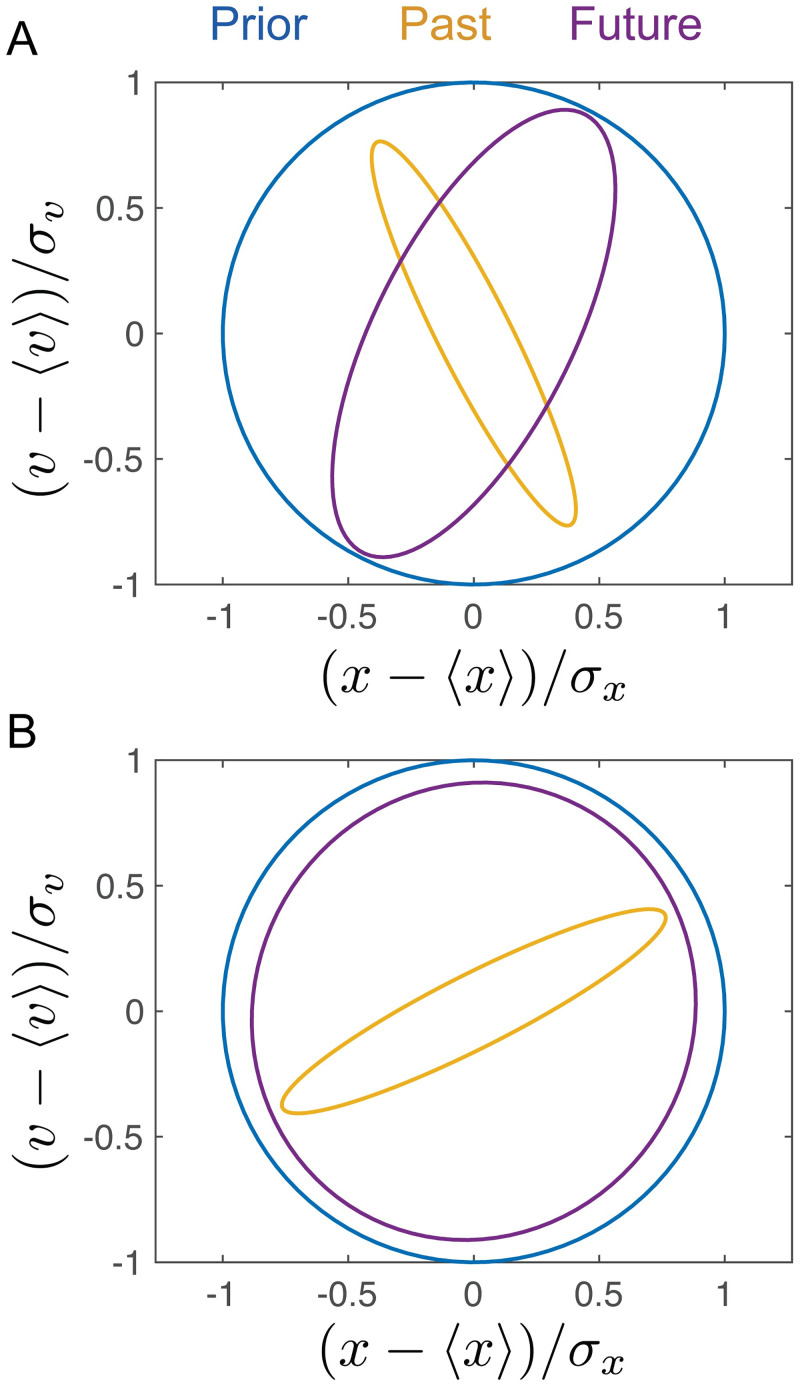
Example of a sub-optimal compression. An optimally predictive, compressed representation, in panel (a) compared to a suboptimal representation, in panel (b) for a prediction at Δ*t* = 1 in the future, within the underdamped regime (*ζ* = 1/2). We fix the mutual information between the representations and *X*_*t*_ ($I(X_{t};\tilde{X})=3$ bits), but find that, as expected, the suboptimal representation contains significantly less information about the future.

Biological systems might not adapt to each input regime perfectly, nor may they be optimally efficient for every possible kind of input dynamics. We consider what happens when an optimal representation is changed, necessarily making it suboptimal for predicting the future stimulus. We construct a new representation by rotating the optimal solution in the position, velocity plane. We examine the conditional distributions for this suboptimal representation, both about *X*_*t*_, $P(X_{t}|{\tilde{X}}_{\text{suboptimal}})$, and the future, $P(X_{t+\Delta t}|{\tilde{X}}_{\text{suboptimal}})$. For a fixed amount of information about *X*_*t*_, $I\left(X_{t};{\tilde{X}}_{\text{optimal}}\right)=I\left(X_{t},{\tilde{X}}_{\text{suboptimal}}\right)$, we compare the predictive information in the optimal (Fig 5A) and the suboptimal representations (Fig 5B). We examine the choice of parameters in the stimulus dynamics for which encoding position alone is an optimal strategy. We note that encoding velocity with high certainty provides very little predictive power, indicating that encoding velocity and position is not equally important, even for equal compression levels. While the nature of the suboptimal and optimal representations depend on the input dynamics, we see that the encoding schemes discovered by the information bottleneck are, indeed, optimally predictive.

#### 2.1.3 Transferability of a representation

So far, we have described the form that optimal predictive compressions take along the information bottleneck curve for a given *ζ* and Δ*t*. How do these representations translate when applied to other prediction timescales (i.e. can the optimal predictive scheme for near-term predictions help generate long-term predictions, too?) or other parameter regimes of the model? This may be important if the underlying parameters in the external stimulus are changing rapidly in comparison to the adaptation timescales in the encoder, which we imagine to be a biological network. For example, a salamander may, on one hand, need to be able to predict at a timescale relevant for prey catching and predict the dynamics of its prey, while on the other, be able to make predictions at different timescales to avoid predators, and predators may have a different dynamical regime [25, 30]. One possible solution is for the encoder to employ a representation that is useful across a wide range of input statistics. This requires that the predictive power of a given representation is, to some extent, transferrable to other input regimes. To quantify how ‘transferrable’ different representations are, we take an optimal representation from one (*ζ*, Δ*t*) and ask how efficiently it captures predictive information for a different parameter regime, (*ζ*′, Δ*t*′).

**Fig 6:**
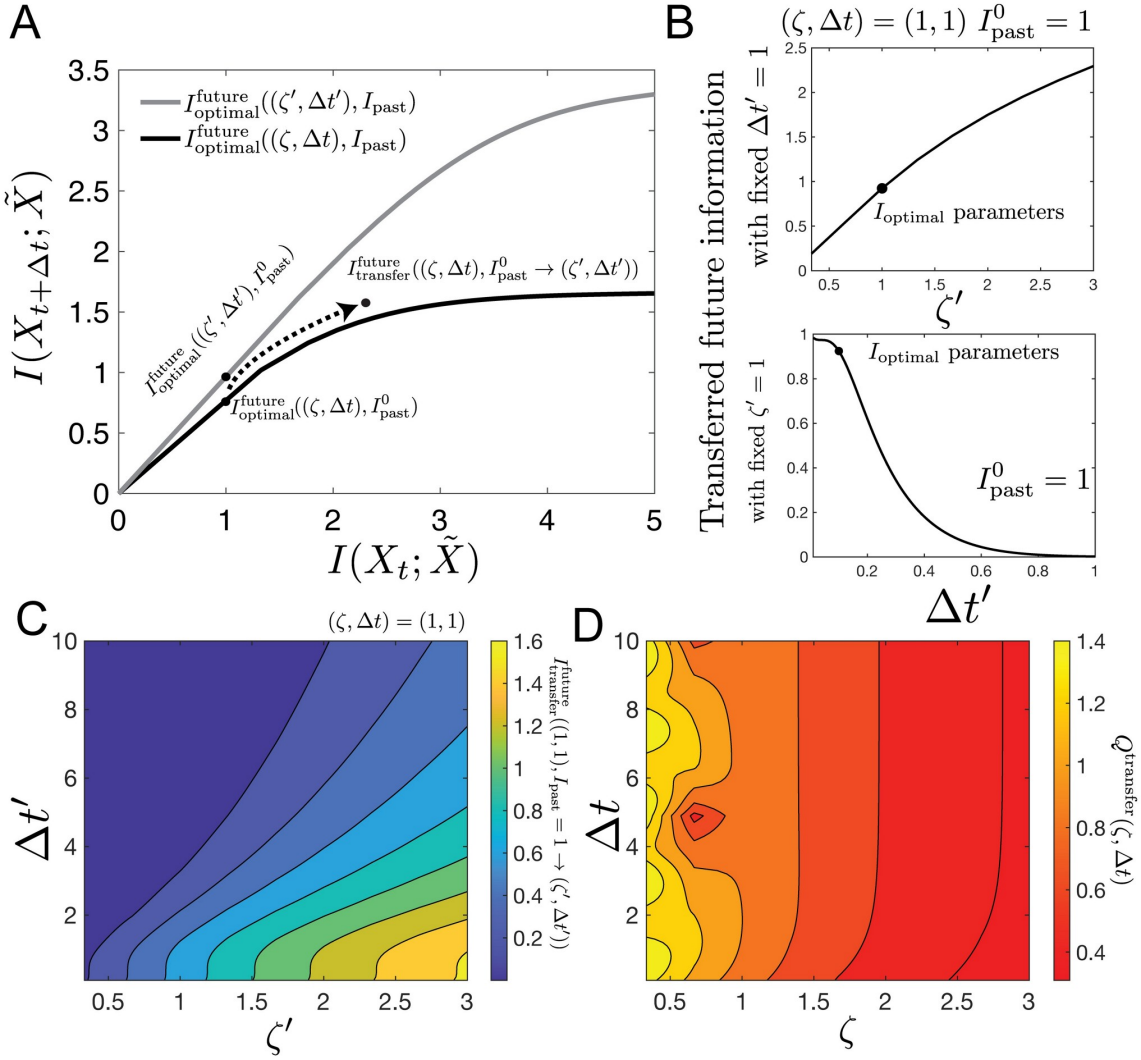
Representations learned on underdamped systems can be transferred to other types of motion, while representations learned on overdamped systems cannot be easily transferred. (a) Here, we consider the information bottleneck bound curve (black) for a stimulus with underlying parameters, (*ζ*, Δ*t*). For some particular level of $I_{\text{past}}=I_{\text{past}}^{0}$, we obtain a mapping, $P(\tilde{X}|X_{t})$ that extracts some predictive information, denoted $I_{\text{optimal}}^{\text{future}}\left(\left(\zeta ,\Delta t\right),I_{\text{past}}^{0}\right)$, about a stimulus with parameters (*ζ*, Δ*t*). Keeping that mapping fixed, we determine the amount of predictive information for dynamics with new parameters (*ζ*′, Δ*t*′), denoted by $I_{\text{transfer}}^{\text{future}}\left(\left(\zeta ,\Delta t\right),I_{\text{past}}^{0}\to \left(\zeta^{\prime },\Delta t^{\prime }\right)\right)$. (b) One-dimensional slices of $I_{\text{transfer}}^{\text{future}}$ in the (*ζ*′, Δ*t*′) plane: $I_{\text{transfer}}^{\text{future}}$ versus *ζ*′ for Δ*t*′ = 1. $I_{\text{past}}^{0}=1$ (top), and versus Δ*t*′ for *ζ*′ = 1. Parameters are set to (*ζ* = 1, Δ*t* = 1), $I_{\text{past}}^{0}=1$. (c) Two-dimensional map of $I_{\text{transfer}}^{\text{future}}$ versus (*ζ*′, Δ*t*′) (same parameters as b). (d) Overall transferability of the mapping. The heatmap of (c) is integrated over *ζ*′ and Δ*t*′ and normalized by the integral of $I_{\text{optimal}}^{\text{future}}\left(\left(\zeta^{\prime },\Delta t^{\prime }\right),I_{\text{past}}\right)$. We see that mappings learned from underdamped systems at late times yield high levels of predictive information for a wide range of parameters, while mappings learned from overdamped systems are not generally useful.

We identify these global strategies by finding the optimal encoder for a stimulus with parameters (*ζ*, Δ*t*) that generates a representation, $P(\tilde{X}|X_{t})$, at some given compression level, *I*_past_. We will label the predictive information captured by this representation $I_{\text{optimal}}^{\text{future}}\left(\left(\zeta ,\Delta t\right),I_{\text{past}}\right)$. We hold the representation fixed and apply it to a stimulus with different underlying parameters (*ζ*′, Δ*t*′) and compute the amount of predictive information the previous representation yields for this stimulus. We call this the transferred predictive information $I_{\text{transfer}}^{\text{future}}\left(\left(\zeta ,\Delta t\right),I_{\text{past}}\to \left(\zeta^{\prime },\Delta t^{\prime }\right)\right)$. We note that $I_{\text{transfer}}^{\text{future}}\left(\left(\zeta ,\Delta t\right),I_{\text{past}}\to \left(\zeta^{\prime },\Delta t^{\prime }\right)\right)$ may sometimes be larger than $I_{\text{optimal}}^{\text{future}}\left(\left(\zeta ,\Delta t\right),I_{\text{past}}\right)$, because changing (*ζ*, Δ*t*) may increase both *I*_past_ and *I*_future_ (see e.g. Fig 6A).

For every fixed (*ζ*, Δ*t*) and *I*_past_, we can take the optimal $\tilde{X}$ and transfer it to a wide range of new *ζ*′’s and timescales, Δ*t*′. For a particular example (*ζ*, Δ*t*), this is shown in Fig 6B. The representation optimized for critical damping is finer-grained than what’s required in the overdamped regime. We can sweep over all combinations of the new *ζ*′’s and Δ*t*′s. What we get, then, is a mapping of $I_{\text{transfer}}^{\text{future}}$ for this representation that was optimized for one particular (*ζ*, Δ*t*) pair across all new (*ζ*′, Δ*t*′)’s. This is shown in Fig 6C, (Fig 6B are just two slices through this surface). This surface gives a qualitative picture the transferability of this particular representation.

To get a quantitative summary of this behavior that we can then compare across different starting points (*ζ*, Δ*t*), we integrate this surface over 1/3 < *ζ*′ < 3, 0.1 < Δ*t*′ < 10, and then normalize by the integral of $I_{\text{optimal}}^{\text{future}}\left(\left(\zeta^{\prime },\Delta t^{\prime }\right),I_{\text{past}}\right)$ over the same surface. This yields an overall transferability measure, *Q*^transfer^(*ζ*, Δ*t*). We report these results in Fig 6D. Representations that are optimal for underdamped systems at late times are the most transferable. This is because generating a predictive mapping for underdamped motion requires some measurement of velocity, which is generally useful for many late-time predictions. Additionally, prediction of underdamped motion requires high precision measurement of position, and that information is broadly useful across all parameters.

### 2.2 History-dependent Gaussian stimuli

In the above analysis, we considered stimuli with temporal correlations that fall off exponentially. However, natural scenes, such as leaves blowing in the wind or bees moving in their hives, are shown to have heavy-tailed statistics [25, 31, 32]. To extend our results to such stimuli, we consider prediction where the statistics of the motion model may feature long-ranged temporal correlations and by increasing the dimensionality of the input and output to the information bottleneck, we demonstrate that the information bottleneck continues to provide useful predictive encoding schemes for such stimuli. We show this through the use of the Generalized Langevin equation [33–35]:

$$\begin{array}{c} \frac{dv}{dt}=-\int_{0}^{t}\frac{\gamma v}{|t-t^{\prime }{|}^{\alpha }}dt-\omega_{0}^{2}x+\xi \left(t\right) \end{array}$$ (15)

$$\begin{array}{c} \frac{dx}{dt}=v \end{array}$$ (16)

Here, we have returned to unscaled definitions of *v*, and *t*. The damping force has a power-law kernel. In order for the system to obey the fluctuation-dissipation theorem, we note that 〈*ξ*(*t*)〉 = 0, and $\langle \xi \left(t^{\prime }\right)\xi \left(t\right)\rangle \propto \frac{1}{|t-t^{\prime }{|}^{\alpha }}$. In this dynamical system, position autocorrelation 〈*x*(*t*)*x*(*t*′)〉 ∼ *t*^−*α*^ and velocity autocorrelation 〈*v*(*t*)*v*(*t*′)〉 ∼ *t*^−*α*−1^ for large *t*.

**Fig 7:**
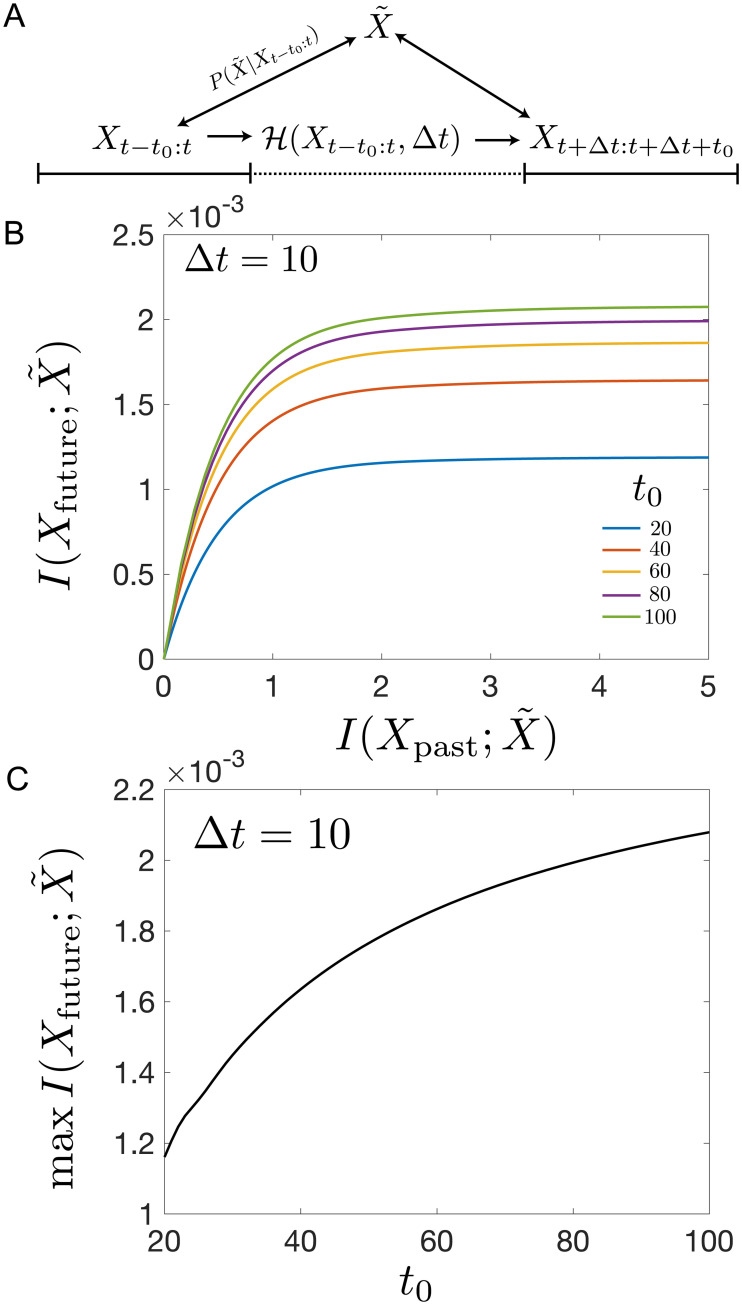
The ability of the information bottleneck Method to predict history-dependent stimuli. (a) The prediction problem, using an extended history and a future. This problem is largely similar to the one set up for the SDDHO but the past and the future are larger composites of observations within a window of time *t*−*t*_0_: *t*, expressed as *X*_past_ for the past and *t* + Δ*t*: *t* + Δ*t* + *t*_0_, expressed as *X*_future_ for the future. (b) Predictive information $I(X_{t+\Delta t:t+\Delta t+t_{0}},\tilde{X})$ with lag Δ*t*. (c) The maximum available predictive information saturates as a function of the historical information used *t*_0_.

The prediction problem is similar to the prediction problem for the memoryless SDDHO, but we now take an extended past, *X*_*t*−*t*_0_:*t*_, for prediction of an extended future, $X_{t+\Delta t:t+\Delta t+t_{0}}$, where *t*_0_ sets the size of the window into the past we consider and the future we predict (Fig 7A). Using the approach described in S1 Text, we compute the optimal representation and determine how informative the past is about the future. The objective function for this extended information bottleneck problem is,

$$\begin{array}{c} L=\min_{P(\tilde{X}|X_{t-t_{0}:t})}I\left(X_{t-t_{0}:t};\tilde{X}\right)-\beta I\left(X_{t+\Delta t:t+\Delta t+t_{0}};\tilde{X}\right). \end{array}$$ (17)

We demonstrate the impacts of the discretization of time in S2. The information bottleneck curves show more predictive information as the prediction process uses more past information (larger *t*_0_ in Fig 7B). Not including any history results in an inability to extract the predictive information. However, for low compression, large *β*, we find that the amount of predictive information that can be extracted saturates quickly as we increase the amount of history, *t*_0_. This implies diminishing returns in prediction for encoding history. Despite the diverging autocorrelation timescale, prediction only functions on a limited timescale and the maximum available prediction information always saturates as a function of *t*_0_ (Fig 7C). These results indicate that efficient coding strategies can enable prediction even in complex temporally correlated environments.

### 2.3 Evolutionary dynamics

Exploiting temporal correlations to make predictions is not limited to vision. Another aspect of the prediction problem appears in the adaptive immune system, where temporal correlations in pathogen evolution may be exploited to help an organism build and maintain immunity in a changing environment. Exploiting these correlations can be done at a population level, in terms of vaccine design [36–39], and has been postulated as a means for the immune system to adapt to future threats [12, 40]. Here, we present efficient predictive coding strategies for the Wright-Fisher model, which is commonly used to describe viral evolution [41]. In contrast to the two models studied so far, Wright-Fisher dynamics are not Gaussian, though they are still Markovian. This implies that predictive information can reside in higher-order moments of the joint distribution, thus the optimal compressed representation variable can no longer be Gaussian. The Wright-Fisher model allows us to explore how the results obtained in the previous sections generalize to non-Gaussian statistics of the past and future distributions. To make this computationally tractable, we will take the representation variable to be discrete, though later allow its cardinality to be large to approximate the continuous solution. There exist methods to approximate continuous compressed representations directly [42–44], though we do not use those here.

**Fig 8:**
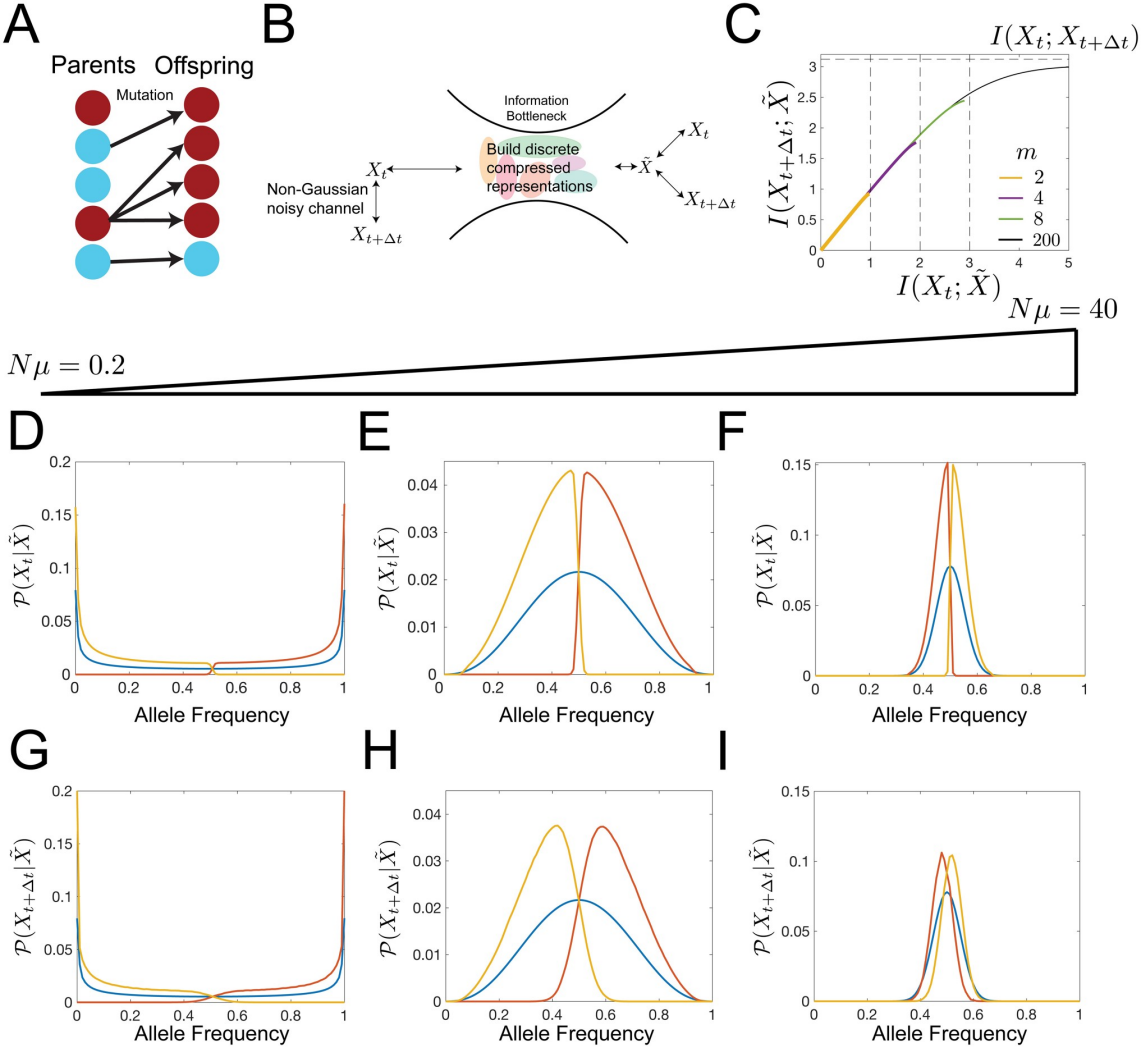
The information bottleneck solution for a Wright Fisher process. (a) The Wright-Fisher model of evolution can be visualized as a population of *N* parents giving rise to a population of *N* offspring. Genotypes of the offspring are selected as a function of the parents’ generation genotypes subject to mutation rates, *μ*, and selective pressures *s*. (b) Information bottleneck schematic with a discrete (rather than continuous) representation variable, $\tilde{X}$. (c) Predictive information as a function of compression level. Predictive information increases with the cardinality, *m*, of the representation variable. The amount of predictive information is limited by log(*m*) (vertical dashed lines) for small *m*, and the mutual information between allele frequencies at time *t* + Δ*t* and time *t*, *I*(*X*_*t*+Δ*t*_;*X*_*t*_) (horizontal dashed line), for large *m*. Bifurcations occur in the amount of predictive information. For small $I(X_{t};\tilde{X})$, the encoding strategies for different *m* are degenerate and the degeneracy is lifted as $I(X_{t};\tilde{X}$) increases, with large *m* schemes accessing higher $I(X_{t};\tilde{X})$ ranges. Parameters: *N* = 100, *Nμ* = 0.2, *Nμ* = 0.2, *Ns* = 0.001, Δ*t* = 1. (d-i) We explore information bottleneck solutions to Wright-Fisher dynamics under the condition that the cardinality of $\tilde{X}$, *m*, is 2 and take *β* to be large enough that $I(X_{t};\tilde{X})\approx 1$, *β* ≈ 4. Parameters: *N* = 100, *Ns* = 0.001, Δ*t* = 1, and *Nμ* = 0.2, *Nμ* = 2, and *Nμ* = 40 (from left to right). (d-f) In blue, we plot the steady state distribution. In yellow and red, we show the inferred historical distribution of alleles based on the observed value of $\tilde{X}$. Note that each distribution is corresponds to roughly non-overlapping portions of allele frequency space. (g-i) Predicted distribution of alleles based on the value of $\tilde{X}$. We observe that as mutation rate increases, the timescale of relaxation to steady state decreases, so historical information is less useful and the predictions becomes more degenerate with the steady state distribution.

Wright-Fisher models of evolution assume a constant population size of *N*. We consider a single mutating site with each individual in the population having either a wild-type or a mutant allele at this site. The allele choice of subsequent generations depends on the frequency of the mutant allele in the ancestral generation at time t, *X*_*t*_, the selection pressure on the mutant allele, *s*, and the mutation rate from the wild-type to the mutant allele and back, *μ*, as depicted as Fig 8A. For large enough *N*, the update rule of the allele frequencies is given through the diffusion approximation interpreted with the Ito convention [45]:

$$\begin{array}{c} \frac{dX_{t}}{dt}=sX_{t}\left(1-X_{t}\right)+\mu \left(1-2X_{t}\right)+\sqrt{X_{t}\left(1-X_{t}\right)/N}\eta \left(t\right), \end{array}$$ (18)

where 〈*η*(*t*)〉 = 0, 〈*η*(*t*)*η*(*t*′)〉 = *δ*(*t* − *t*′). We note that this model is Markovian, so as we did with the SDDHO, we will take the historical variable to be *X*_*t*_ and the future variable to be *X*_*t*+Δ*t*_. Details are given in S3 Text. Extending the timescale of the representation of the past will not confer additional predictive information.

For this model, defining the representation $\tilde{X}$ as a noisy linear transformation of *X*_*t*_, the allele frequency at time *t*, as we did for the Gaussian case in S1 Text. Eq 1 does not capture all of the dependences between the past and future allele frequencies, because correlations exist beyond second order. This arises because of the non-linear form of Eq 18. Instead, we determine the mapping of *X*_*t*_ to $\tilde{X}$ numerically using the Blahut-Arimoto algorithm [46, 47]. In general, for a discrete representation variable $\tilde{X}$, the true cardinality of $\tilde{X}$ is unknown for a given *β*. Our approach is to first fix the cardinality of $\tilde{X}$ to a given value *m* (Fig 8C) and compute the information curve for the given *m* by sweeping over *β*. We then repeat this for larger values of *m*. We note that for small *β*, the solutions for different values of *m* are degenerate, while at higher values of *β*, bifurcations emerge between encoding schemes for the solutions with cardinality *m* and *m* − 1. This is because the true cardinality of the optimal solution undergoes transitions to higher and higher values as *β* increases [14]. The discreteness of $\tilde{X}$ results in each realization of the representation tiling a distinct part of frequency space. This encoding scheme can be thought of a different types of immune defenses: innate, adaptive, and different lymphocyte phenotypes acting at different stages or for different types of immune responses [48]. Accordingly, *m* would correspond to the number of distinct cell types mobilized against pathogens of various frequencies. The concept of discrete tiling of space is also analogous to ideas of immune coverage, whereby a finite number of distinct antigen receptors cover the entire “shape space” of possible antigens [49]. However, to make this analogy more precise would require to study an effective theory of phenotypic evolution [50].

We first consider the example with *m* = 2 representations. In the weak-mutation, weak-selection limit (*Nμ*, *Ns* ≪ 1), the steady state probability distribution of allele frequencies,

$$\begin{array}{c} P_{s}\left(X\right)\propto {\left[X(1-X)\right]}^{N\mu -1}e^{NsX} \end{array}$$ (19)

(blue line in Fig 8D) is peaked around the frequency boundaries, indicating that at long times, an allele either fixes or goes extinct. In this case, one value of the representation variable corresponds to the range of high allele frequencies and the other corresponds to low allele frequencies (Fig 8D, yellow and red lines). These encoding schemes can be used to make predictions, whether it be by an observer or the immune system, via determining the future probability distribution of the alleles conditioned on the value of the representation variables, $P(X_{t+\Delta t}|\tilde{X})$. We present these predictions in Fig 8G. The predictive information conferred by the representation variable is limited by the information it has about *X*_*t*_ as in the Gaussian case (Fig 8C).

For larger mutation rates, the steady state distribution becomes centered around the equal probability of observing either one of the two alleles, but the two representation variables still cover the frequency domain in way that minimizes overlap (Fig 8E and 8F). We observe a sharp drop in $P(X_{t}|\tilde{X})$ at the boundary between the two representations. The future distribution of allele frequencies in this region (Fig 8H and 8I), however, displays large overlap. The degree of this overlap increases as the mutation rate gets larger, suggesting prediction is harder in the strong mutation limit. The optimal encoding of the distribution of *X*_*t*_ biases the representation variable towards frequency space regions with larger steady state probability mass.

**Fig 9:**
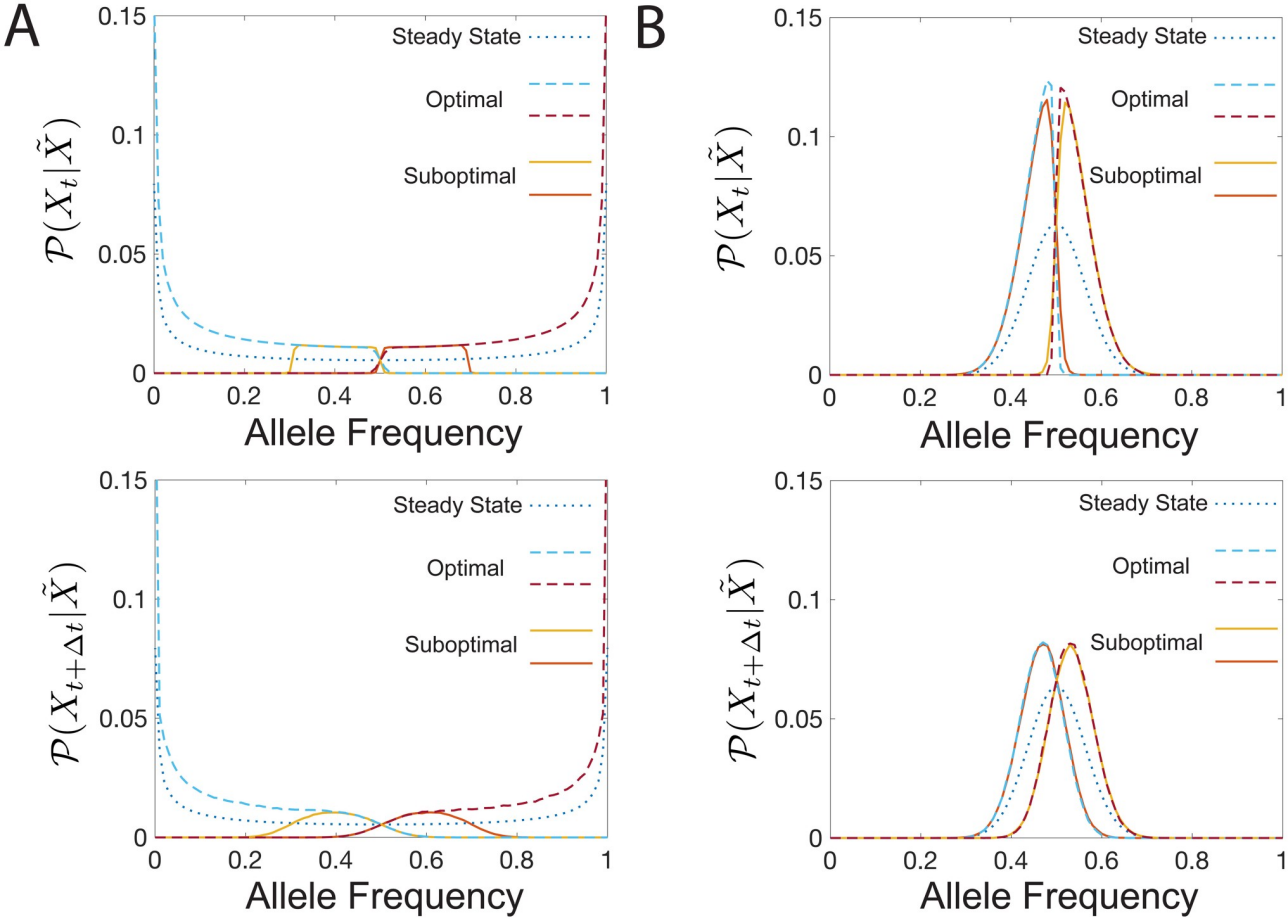
Transferability of prediction schemes in Wright-Fisher dynamics. We transfer a mapping, $P(\tilde{X}|X_{t})$, trained on one set of parameters and apply it to another. We consider transfers between two choices of mutability, *Nμ*_1_ = 0.2 (low) and *Nμ*_2_ = 20 (high), with *N* = 100, *Ns* = 0.001, Δ*t* = 1. The dotted line is the steady state allele frequency distribution, the solid lines are the transferred representations, and the dashed lines are the optimal solutions. The top panels correspond to the distributions of *X*_*t*_ and the bottom panels correspond to distributions of *X*_*t*+Δ*t*_. (a) Transfer from high to low mutability. Optimal information values: $I_{\text{optimal}}^{\text{past}}=0.98$ and $I_{\text{optimal}}^{\text{future}}=0.93$; transferred information values: $I_{\text{transfer}}^{\text{past}}\left(\left(N\mu_{2}\right),I_{\text{past}}=0.92\to \left(N\mu_{1}\right)\right)=0.14$ and $I_{\text{transfer}}^{\text{future}}\left(\left(N\mu_{2}\right),I_{\text{past}}=0.92\to \left(N\mu_{1}\right)\right)=0.05$. Representations learned on high mutation rates are not predictive in the low mutation regime. (b) Transfer from low to high mutability. Optimal information values: $I_{\text{optimal}}^{\text{past}}=0.92$ and $I_{\text{optimal}}^{\text{future}}=0.92$ and $I_{\text{optimal}}^{\text{future}}=0.28$. Transferred information values: $I_{\text{transfer}}^{\text{past}}\left(\left(N\mu_{1}\right),I_{\text{past}}=0.98\to \left(N\mu_{2}\right)\right)=0.79$ and $I_{\text{transfer}}^{\text{future}}\left(\left(N\mu_{1}\right),I_{\text{past}}=0.98\to \left(N\mu_{2}\right)\right)=0.27$. Transfer in this direction yields good predictive informations.

**Fig 10:**
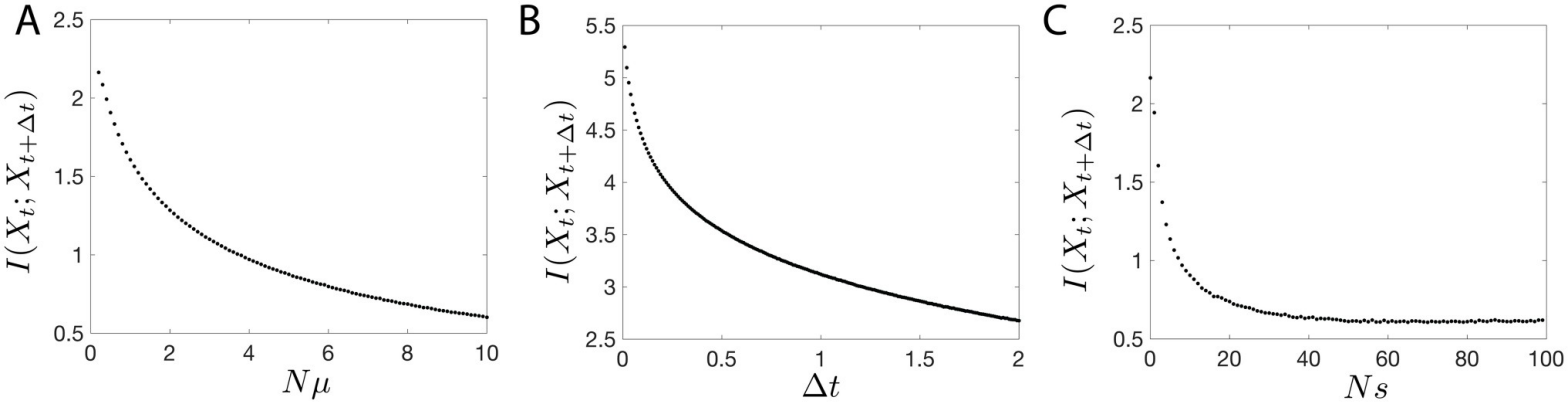
Amount of predictive information in the Wright Fisher dynamics as a function of model parameters. (a-c), Value of the asymptote of the information bottleneck curve, *I*(*X*_*t*_;*X*_*t*+Δ*t*_) with: (a) *N* = 100, *Ns* = 0.001, Δ*t* = 1 as a function of *μ*; (b) *N* = 100, *Nμ* = 0.2, *Ns* = 0.001 as a function of Δ*t*; and (c) *N* = 100, *Nμ* = 0.2, and Δ*t* = 1 as a function of *s*.

In Fig 9, we explore the consequence of transferring a mapping, $P(\tilde{X}|X_{t})$, from a high mutation model to a low mutation model and vice versa. We observe that the weak mutation representation is more transferrable than the strong mutation representation. One reason for this is that the strong mutation limit provides little predictive information, as seen in Fig 10A. In addition, high mutation representations focus on *X* = 1/2, while the population more frequently occupies allele frequencies near 0 and 1 in other regimes. Comparatively, representations learned on weak mutation models can provide predictive information, because they cover more evenly the spectrum of allele frequencies.

We can extend the observations in Fig 8 to see how the predictive information depends on the strength of the selection and mutation rates (Fig 10A and 10C). Prediction is easiest in the weak mutation and selection limit, as population genotype change occur slowly and the steady state distribution is localized in one regime of the frequency domain. For evolutionary forces acting on faster timescales, prediction becomes harder since the relaxation to the steady state is fast. Although the mutation result might be expected, the loss of predictive information in the high selection regime seems counterintuitive: due to a large bias between one of the two alleles evolution appears reproducible and “predictable” in the high selection limit. This bias renders the allele state easier to guess but this is not due to information about the initial state. The mutual information-based measure of predictive information used here captures a reduction of entropy in the estimation of the future distribution of allele frequencies due to conditioning on the representation variable. When the entropy of the future distribution of alleles *H*(*X*_*t*+Δ*t*_) is small, the reduction is small and predictive information is also small. As expected, predictive information decreases with time Δ*t*, since the state *X*_*t*_ and *X*_*t*+Δ*t*_ decorrelate due to noise (Fig 10B).

**Fig 11:**
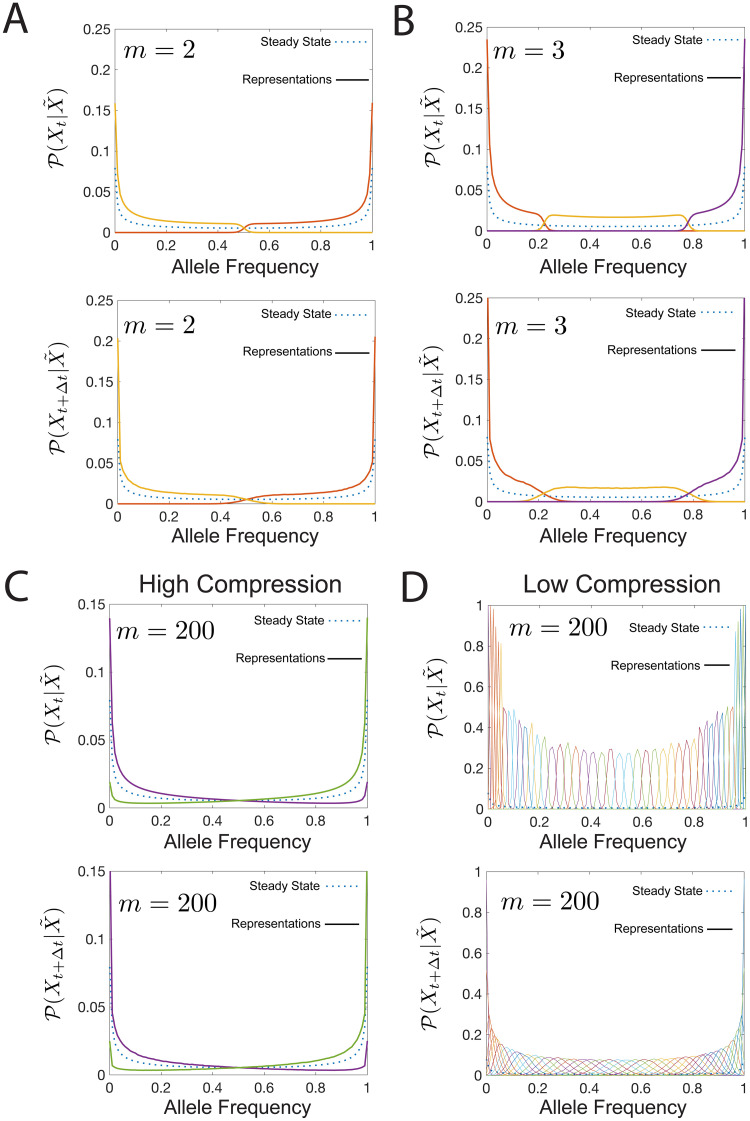
Encoding schemes with *m* > 2 representation variables. The steady state is plotted as a dotted line and the representation for each realization of the value of $\tilde{X}$ are plotted as solid lines. The representations which carry maximum predictive information for (a) *m* = 2 at $I\left(X_{t};\tilde{X}\right)\approx \log\left(m\right)=1$ bit, and (b) *m* = 3 at $I\left(X_{t};\tilde{X}\right)\approx \log\left(m\right)\approx 1.5$ bits. The optimal representations at large *m* tile space more finely and have higher predictive information. The optimal representations for *m* = 200 at fixed *β* = 1.01 ($I(X_{t};\tilde{X})=0.28$, $I(X_{t+\Delta t};\tilde{X})=0.27$) (c) and *β* = 20 ($I(X_{t};\tilde{X})=2.77$, $I(X_{t+\Delta t};\tilde{X})=2.34$). (d) At low $I(X_{t};\tilde{X})$, many of the representations are redundant and do not confer more predictive information than the *m* = 2 scheme. A more explicit comparison is given in S3 Fig. At high $I(X_{t};\tilde{X})$, the degeneracy is lifted. All computations done at *N* = 100, *Nμ* = 0.2, *Ns* = 0.001, Δ*t* = 1.

So far we have discussed the results for *m* = 2 representations. As we increase the tradeoff parameter, *β* in Eq 1, the amount of predictive information increases, since we retain more information about the the allele frequency at time *t*. However, at high *β* values the amount of information the representation variable can hold saturates, and the predictive information reaches a maximum value (1 bit for the *m* = 2 yellow line in Fig 10A). Increasing the number of representations *m* to 3 increases the range of accessible information the representation variable has about the past *I*(*X*_*t*_;*X*), increasing the range of predictive information (purple line in Fig 8C)). Comparing the *m* = 2 and *m* = 3 representations for maximum values of *β* for each of them (Fig 11A and 11B), shows that larger numbers of representations tile allele frequency space more finely, allowing for more precise encodings of the past and future distributions. The maximum amount of information about the past goes as log(*m*) (Fig 8C). The predictive information curves for different *m* values are the same, until the branching point ≲ log(*m*) for each *m* (Fig 8C).

We analyze the nature of this branching by taking *m* ≫ 1, *m* = 200 (Fig 11C and 11D). At small *β* (and corresponding small $I(X_{t};\tilde{X})$) the optimal encoding scheme is the same if we had imposed a small *m* (Fig 11C), with additional degenerate representations (S3 Fig). By increasing *β* (and $I(X_{t};\tilde{X})$), the degeneracy is lifted and additional representation cover non-overlapping regimes of allele frequency space. This demonstrates the existence of a critical *β* for each predictive coding scheme, above which *m* needs to be increased to extract more predictive information and below which additional values of the representation variable encode redundant portions of allele frequency space. While we do not estimate the critical *β*, approaches to estimating them are presented in [51, 52].

The *m* = 200 encoding approximates the continuous $\tilde{X}$ representation. In the high $I(X_{t};\tilde{X})$ limit, the *m* = 200 encoding gives precise representations (i.e. with low variability in $P(X_{t}|\tilde{X})$) in regions of allele frequency space with high steady state distribution values, and less precise representations elsewhere (Fig 11D top panel and S4 Fig). This dependence differs from the Gaussian case, where the uncertainty of the representation is independent of the encoded value. The decoding distributions $P(X_{t}|\tilde{X})$ are also not Gaussian. This encoding builds a mapping of internal response to external stimuli, by tiling the internal representation space of external stimuli in a non-uniform manner. These non-uniform frequency tilings are similar to Laughlin’s predictions for maximally informative coding in vision [2], but with the added constraint of choosing the tiling to enable the most informative predictions.

## 3 Discussion

We have demonstrated that the information bottleneck method can be used to construct predictive encoding schemes for a variety of biologically-relevant dynamic stimuli. The approach described in this paper can be used to make predictions about the underlying encoding schemes used by biological systems that are compelled by their behavioral and fitness constraints to make predictions. These results thus provide experimentally testable hypotheses. The key principle is that not all input dimensions are equally relevant for prediction; information encoding systems must be able to parse which dimensions are relevant when coding capacity is small relative to the available predictive information. Hence, the biological (or engineered) system must navigate a tradeoff between reducing the overall uncertainty in its prediction while only being able to make measurements with some fixed uncertainty.

It may not always be the case, experimentally, that a system uses an optimal encoding for prediction of a particular motion stimulus. When the stimulus nonetheless falls within the natural scene input repertoire for the organism, we hypothesize that biological systems may use a best-compromise predictive encoding of their inputs because that need to operate flexibly across a wide range of different input statistics. We provide a transferability metric, *Q*, which quantifies how useful a particular scheme is across other dynamic regimes and prediction timescales, that can be used to experimentally predict what the best-compromise predictive encoding scheme is in cases where a biological system needs to be flexible. We observe that a compromise between representing position and velocity of a single object provides a good, general, predictor for a large set of input behaviors. When adaptation is slower than the timescale over which the environment changes, such a compromise might be beneficial to the biological system. On the other hand, if the biological encoder can adapt, the optimal predictive encoder for those particular dynamics is the best encoder. We have provided a fully-worked set of examples of what those optimal encoders look like for a variety of parameter choices. The dynamics of natural inputs to biological systems could be mapped onto particular points in these dynamics, providing a hypothesis for what optimal prediction would look like in that system.

We also explored the ability to predict more complex, non-Markovian dynamics. We asked about the usefulness of storing information about the past in the presence of power-law temporal correlations. The optimal information bottleneck solution showed fast diminishing returns as it was allowed to dig deeper and deeper into the past, suggesting that simple encoding schemes with limited temporal span have good predictive power even in complex correlated environments.

Superficially, our framework may seem similar to a Kalman filter [53]. There are few major differences in this approach. Kalman filtering algorithms have been used to explain responses to changes in external stimuli in biological system [54]. In this framework, the Kalman filters seek to maximize information by minimizing the variance in estimating the true coordinates of an external input. The estimate is, then, a prediction of the next time step, and is iteratively updated. Our information bottleneck approach extracts past information, but explicitly includes another constraint: resource limitations. The tuning of *I*_past_ is the main difference between our approach and a Kalman filter. Another major difference is that we do not assume the underlying encoder has any explicit representation of the ‘physics’ of the input. There is no internal model of the input stimulus, apart from our probabilistic mapping from the input to our compressed representation of that input. A biological system could have such an internal model, but that would add significant coding costs that would have to be treated by another term in our framework to draw a precise equivalence between the approaches. We show in the S1 Fig that the Kalman filter approach is not as efficient, in general, as the predictive information bottleneck approach that we present here.

Our results on systems with Wright-Fisher input dynamics reveal that discrete representations that tile input space are optimally predictive encoders. Although we impose discrete internal representations, their non-overlapping character remains even it the limit of a large number of representations. These kinds of solutions are reminiscent of the Laughlin solution for information maximization of input and output in the visual system given a nonlinear noisy channel [2], in which the input space is covered proportionally to the steady state distribution at a given frequency, in chunks given by the size of the noise in the system. Tiling solutions have also been described when optimizing information in gene regulatory networks with nonlinear input-output relations, when one input regulates many gene outputs [55]. In this case each gene was expressed in a different region of the input concentration domain. Similarly to our example, where the lifting the degeneracy between multiple representations covering the same frequency range allows for the prediction of more information about the future, lifting the degeneracy between different genes making the same readout, increases the transmitted information between the input concentration and the outputs. More generally, discrete tiling solutions are omnipresent in information optimization problems with boundaries [56, 57].

Biologically, predicting evolutionary dynamics is a different problem than predicting motion. Maybe the accuracy of prediction matters less, while covering the space of potentially very different inputs is important. In our simple example, this is best seen in the strong mutation limit where the mutant allele either fixes or goes extinct with equal probability. In this case, a single Gaussian representation cannot give a large values of predictive information. A discrete representation, which specializes to different regions of input space, is a way to maximize predictive power for very different inputs. It is likely that these kinds of solutions generalize to the case of continuous, multi-dimensional phenotypic spaces, where discrete representations provides a way for the immune system to hedge its bets against pathogens by covering the space of antigen recognition [28]. The tiling solution that appears in the non-Gaussian solution of the problem is also potentially interesting for olfactory systems. The number of odorant molecules is much larger than odor receptors [58, 59], which can be thought of as representation variables that cover the phenotypic input space of odorants. The predictive information bottleneck solution gives us a recipe for covering space, given a dynamical model of evolution of the inputs.

The results in the non-Gaussian problem are different than the Gaussian problem in two important ways: the encoding distributions are not Gaussian (e.g. Fig 8D and 8E), and the variance of the encoding distributions depends on the the value of $P(X_{t}|\tilde{X})$ (Fig 11D). These solutions offer more flexibility for internal encoding of external signals.

The information bottleneck approach has received a lot of attention in the machine learning community lately, because it provides a useful framework for creating well-calibrated networks that solve classification problems at human-level performance [15, 42, 60]. In these deep networks, variational methods approximate the information quantities in the bottleneck, and have proven their practical utility in many machine learning contexts. These approaches do not always provide intuition about how the networks achieve this performance and what the information bottleneck approach creates in the hidden encoding layers. Here, we have worked through a set of analytically tractable examples, laying the groundwork for building intuition about the structure of information bottleneck solutions and their generalizations in more complex problems.

In summary, the problem of prediction, defined as exploiting correlations about the past dynamics to anticipate the future state comes up in many biological systems from motion prediction to evolution. This problem can be formulated in the same way, although as we have shown, the details of the dynamics matter for how best to encode a predictive representation and maximize the information the system can retain about the future state. Dynamics that results in Gaussian propagators is most informatively predicted using Gaussian representations. However non-Gaussian propagators introduce disjoint non-Gaussian representations that are nevertheless predictive.

By providing a set of dissected solutions to the predictive information bottleneck problem, we hope to show that not only is the approach feasible for biological encoding questions, it also illuminates connections between seemingly disparate systems (such as visual processing and the immune system). In these systems the overarching goal is the same, but the microscopic implementation might be very different. Commonalities in the optimally predictive solutions as well as the most generalizable ones can provide clues about how to best design experimental probes of this behavior, at both the molecular and cellular level or in networks.

## Methods

Computational methods are as described in the Results and in S1–S3 Text.

## Supporting information

S1 Text Computing the optimal representation for jointly Gaussian past-future distributions. We present the results of [16], which is a a derivation for the solution to the information bottleneck in the limit of jointly Gaussian variables. This formalism is used throughout the text to analytically produce the results presented.
(PDF)

S2 Text The stochastically driven damped harmonic oscillator. We provide full derivations for our results involving the harmonic oscillator, including extensions to generalized frictional kernels. We also provide some comparison to another popular scheme, the Kalman filter.
(PDF)

S3 Text Wright-Fisher dynamics. We provide some detail about the parameters in our simulation for Wright-Fisher dynamics and a short derivation for the maximum amount of encoded information for a given *m*.
(PDF)

S1 Fig Kalman filtering schemes are not efficient coders for a given channel capacity. We compare the amount of information conferred about the future for a given encoding level and find that Kalman Filter-based approaches do not maximize the amount of predictive information conferred, suggesting they are not efficient predictive coding schemes.
(TIF)

S2 Fig We plot the information curve for Δ*t* = 10, *t*_0_ = 20 for different values of *dt*. We note that there are diminishing returns for increasingly small *dt*. However, we cannot make *dt* arbitrarily small, as this introduces numerical errors.
(TIF)

S3 FigThe optimal $P(X_{t}|\tilde{X})$ and $P(X_{t+\Delta t}|\tilde{X})$ for Wright Fisher dynamics with *N* = 100, *Nμ* = 0.2, *Ns* = 0.001, Δ*t* = 1 with information bottleneck parameters *β* = 1.01 ($I(X_{t};\tilde{X})=0.27$) for *m* = 2 (a) and *m* = 200 (b).Many representations are degenerate in the *m* = 200 in this limit. The encoding schemes for *m* = 2 versus *m* = 200 are nearly identical for this small $I(X_{t};\tilde{X})$ limit.(TIF)

S4 FigMean (left) and variance (right) of the past allele frequency *X*_*t*_ conditioned on the (categorical) representation variable $\tilde{X}$ (left), for the information bottleneck solution of the Wright-Fisher dynamics with *m* = 200, *N* = 100, *Nμ* = 0.2, *Ns* = 0.001, *β* = ∞.The standard deviation is not constant: it is smaller where the prior probability of *X*_*t*_ is large.(TIF)
