# Supplementary material for: Optimal prediction with resource constraints using the information bottleneck
Source: PLoS Comput Biol. 2021 Mar 8;17(3):e1008743. doi: 10.1371/journal.pcbi.1008743 (PMC7971903; doi:10.1371/journal.pcbi.1008743)
Supplement: S1 Text — We present the results of [16], which is a a derivation for the solution to the information bottleneck in the limit of jointly Gaussian variables. This formalism is used throughout the text to analytically produce the results presented. (PDF) [file pcbi.1008743.s001.pdf]

S1 Text: Computing the optimal representation for jointly Gaussian past-future distributions

February 3, 2021

We reproduce Chechik et al. [1] to show the analytic construction of the optimally predictive representation variable, $\tilde{X}$, when the input and output variables are jointly Gaussian. The input is $X_t \sim \mathcal{N}(0, \Sigma_{X_t})$ and the output is $X_{t+\Delta t} \sim \mathcal{N}(0, \Sigma_{X_{t+\Delta t}})$. The joint distribution of X_t and $X_{t+\Delta t}$ is Gaussian. To construct the representation, we take a noisy linear transformation of X_t to define $\tilde{X}$

$$\tilde{X} = A_\beta X_t + \xi. \tag{1}$$

Here, A_β is a matrix whose elements are a function of β, the tradeoff parameter in the information bottleneck objective function between compressing, in our case, the past while retaining information about the future. ξ is a vector of dimension $\dim(X_t)$. The entries of ξ are Gaussian-distributed random numbers with 0 mean and unit variance. Because the joint distribution of the past and the future is Gaussian, to capture the dependencies of $X_{t+\Delta t}$ on X_t we can use a noisy linear transform of X_t to construct a representation variable that satisfies the information bottleneck objective function [1].

We compute A_β by first computing the left eigenvectors and the eigenvalues of the regression matrix, $\Sigma_{X_t|X_{t+\Delta t}}\Sigma_{X_t}^{-1}$. Here, $\Sigma_{X_t|X_{t+\Delta t}}$ is the covariance matrix of the probability distribution of $\mathcal{P}(X_t|X_{t+\Delta t})$. These eigenvector–eigenvalue pairs satisfy the following relation

$$v_i^T \Sigma_{X_t|X_{t+\Delta t}} \Sigma_{X_t}^{-1} = \lambda_i v_i^T. \tag{2}$$

(We are taking v_i^T to be a row vector, rather than a column vector.)

The matrix, A_β, is then given by

$$A_\beta = \begin{bmatrix} \alpha_1 v_1^T \\ \alpha_2 v_2^T \\ \vdots \end{bmatrix}. \tag{3}$$

α_i are scalar values given by

$$\begin{aligned} \alpha_i =& \sqrt{\frac{\beta(1-\lambda_i)-1}{\lambda_i v_i^T \Sigma_{X_t} v_i}} && \text{if } \beta > \frac{1}{1-\lambda_i} \\ \alpha_i =& 0 && \text{otherwise.} \end{aligned} \tag{4}$$

The α_i define the dimensionality of the most informative representation variable, $\tilde{X}$. The dimension of $\tilde{X}$ is the number of non-zero α_i. The optimal dimension for a given β is, at most, equal to the dimension of $X_{t+\Delta t}$. The set of values, $\{\beta_{c_i} | \beta = 1/(1-\lambda_i)\}$, can be thought of as critical values, as each β_{c_i} triggers the inclusion of the ith left eigenvector into the optimal $\tilde{X}$. The critical values depend strongly on the particular statistics of the input and output variable, so they may be different as the parameters that generate X change.

To compute the information about the past and future contained in $\tilde{X}$, we compute $\mathcal{P}(X_t|\tilde{X})$ and $\mathcal{P}(X_{t+\Delta t}|\tilde{X})$. These distributions are Gaussian. The mean of each distribution corresponds to the encoded value of X_t and $X_{t+\Delta t}$. The variance corresponds to the uncertainty, or entropy, in this estimate. To compute the variance, we need the variance of $\tilde{X}$

$$\Sigma_{\tilde{X}} = \langle \tilde{X}^T \tilde{X} \rangle = \langle \tilde{X}^T A_\beta^T A_\beta \tilde{X} \rangle + \langle \xi^T \xi \rangle, \tag{5}$$

where the excluded terms are zero. Recalling the definition of ξ, we can simplify this expression to yield

$$\Sigma_{\tilde{X}} = A_\beta \Sigma_{X_t} A_\beta^T + I_2. \tag{6}$$

Here, I_2 is the identity matrix. To compute the mutual information quantities, we use the following equations,

$$\begin{aligned} I(X_t;\tilde{X}) &= \frac{1}{2}\log_2(|A_\beta \Sigma_{X_t} A_\beta^T + I_2|), \\ I(X_{t+\Delta t};\tilde{X}) &= I(X_t;\tilde{X}) - \frac{1}{2}\sum_{i=1}^{n(\beta)} \log_2(\beta(1-\lambda_i)), \end{aligned} \tag{7}$$

where $n(\beta)$ corresponds to the number of dimensions included in A_β. We also need the cross covariances between $\tilde{X}$ and X_t and between $\tilde{X}$ and $X_{t+\Delta t}$, which are particularly useful for visualizing the optimal predictive encoding. To obtain these matrices, we use

$$\begin{aligned} \Sigma_{\tilde{X}X_t} &= A_\beta \Sigma_{X_t} \\ \Sigma_{\tilde{X}X_{t+\Delta t}} &= A_\beta \Sigma_{X_{t+\Delta t}X_t}. \end{aligned} \tag{8}$$

We can use these results and the Schur complement formula to obtain

$$\begin{aligned} \Sigma_{X_t|\tilde{X}} &= \Sigma_{X_t} - \Sigma_{X_t\tilde{X}} \Sigma_{\tilde{X}}^{-1} \Sigma_{X_t\tilde{X}}^T \\ \Sigma_{X_{t+\Delta t}|\tilde{X}} &= \Sigma_{X_{t+\Delta t}} - \Sigma_{X_{t+\Delta t}\tilde{X}} \Sigma_{\tilde{X}}^{-1} \Sigma_{X_{t+\Delta t}\tilde{X}}^T. \end{aligned} \tag{9}$$

References

1. Chechik G, Globerson A, Tishby N, Weiss Y. Information Bottleneck for Gaussian Variables. In: Thrun S, Saul LK, Schölkopf B, editors. Advances in Neural Information Processing Systems 16. MIT Press; 2004. p. 1213–1220. Available from: http://papers.nips.cc/paper/2457-information-bottleneck-for-gaussian-variables.pdf.
