# Supplementary material for: Optimal prediction with resource constraints using the information bottleneck
Source: PLoS Comput Biol. 2021 Mar 8;17(3):e1008743. doi: 10.1371/journal.pcbi.1008743 (PMC7971903; doi:10.1371/journal.pcbi.1008743)
Supplement: S2 Text — We provide full derivations for our results involving the harmonic oscillator, including extensions to generalized frictional kernels. We also provide some comparison to another popular scheme, the Kalman filter. (PDF) [file pcbi.1008743.s002.pdf]

# S2 Text: The Stochastically Driven Dampled Harmonic Oscillator

February 3, 2021

## A Harmonic Oscillator Model With No Memory

We begin by considering a mass attached to a spring undergoing viscous damping. The mass is being kicked by thermal noise. This mechanical system is largely called the stochastically driven damped harmonic oscillator (SDDHO). A simple model for its position and velocity evolution is given by

$$
\begin{aligned}
m\frac{dv}{dt} &= -\Gamma v(t) - kx + (2k_BT\Gamma)^{1/2}\xi(t) \\
\frac{dx}{dt} &= v.
\end{aligned} \tag{1}
$$

We use the redefined variables presented in the main text Equations $2-9$ to rewrite the equations as

$$
\begin{aligned}
\frac{dv}{dt} &= -\frac{x}{4\zeta^2} - v + \frac{\xi(t)}{\sqrt{2}\zeta} \\
\frac{dx}{dt} &= v.
\end{aligned} \tag{2}
$$

There are now two key parameters to explore: $\zeta$ and $\Delta t$. There are three regimes of motion described by this model. The overdamped regime occurs when $\zeta > 1$. In this regime of motion, the mass, when perturbed from its equilibrium position, relaxes back to its equilibrium position slowly. The underdamped regime occurs when $\zeta < 1$. In this regime of motion, when the mass is perturbed from its equilibrium position, it oscillates about its equilibrium position with an exponentially decaying amplitude. At $\zeta = 1$, we are in the critically damped regime of motion; in this regime, when the mass is perturbed from equilibrium, it returns to equilibrium position as quickly as possible without any oscillatory behavior.

To apply the information bottleneck method to this system, we need to compute the following covariance and cross covariance matrices: $\Sigma_{X_t}$, $\Sigma_{X_{t+\Delta t}}$, and $\Sigma_{X_tY_{t+\Delta t}}$. We note that because the defined motion model is stationary in time, $\Sigma_{X_t} = \Sigma_{X_{t+\Delta t}}$. Using the procedure given in Flyvbjerg et. al. [1], we can compute the requisite autocorrelations to describe the cross-covariance matrix, $\Sigma_{X_tX_{t+\Delta t}}$.

We begin by using the equipartition theorem that states that

$$
\begin{aligned}
\langle x_0^2 \rangle &= 1 \\
\langle x_0 v_0 \rangle &= 0 \\
\langle v_0^2 \rangle &= \frac{1}{4\zeta^2}.
\end{aligned} \tag{3}
$$

The covariance matrices are symmetric, so we can use these values to define the elements of $\Sigma_{X_t}$. We then obtain expressions for $\Sigma_{X_t X_{t+\Delta t}}$

$$\Sigma_{X_t X_{t+\Delta t}} = \exp\left(-\frac{\Delta t}{2}\right)\begin{bmatrix}\cos(\omega\Delta t) + \frac{\sin(\omega\Delta t)}{2\omega} & -\frac{\sin(\omega\Delta t)}{4\zeta^2\omega} \\ \frac{\sin(\omega\Delta t)}{4\zeta^2\omega} & \frac{\cos(\omega\Delta t)}{4\zeta^2} - \frac{\sin(\omega\Delta t)}{8\omega\zeta^2}\end{bmatrix} \tag{4}$$

where we have defined $\omega^2 = \frac{1}{4\zeta^2} - \frac{1}{4}$. An alternative approach for the derivation of the above correlation values by methods of Laplace transforms can be found in Sandev et. al. [2].

To construct the optimal representation for prediction, we need the conditional covariance matrices, $\Sigma_{X_t|X_{t+\Delta t}}$ and $\Sigma_{X_{t+\Delta t}|X_t}$. This can be computed using the Schur complement formula to yield

$$\begin{aligned}\Sigma_{X_t|X_{t+\Delta t}} &= \Sigma_{X_t} - \Sigma_{X_t X_{t+\Delta t}}\Sigma_{X_t}^{-1}\Sigma_{X_t X_{t+\Delta t}}^T \\ \Sigma_{X_{t+\Delta t}|X_t} &= \Sigma_{X_t} - \Sigma_{X_t X_{t+\Delta t}}^T\Sigma_{X_t}^{-1}\Sigma_{X_t X_{t+\Delta t}}\end{aligned} \tag{5}$$

We provide a graphical representation of these distributions in Fig 2B (main text). These graphical representations correspond to the contour inside which $\sim 68\%$ of observations are observed (i.e. one standard deviation from the mean).

# B Applying the information bottleneck Solution

To apply the information bottleneck solution, we construct the matrix, $\Sigma_{X_t|X_{t+\Delta t}}\Sigma_{X_t}^{-1}$, and find its eigenvalues and eigenvectors. The left eigenvectors of the matrix will be denoted by the columns of a new matrix, $w$, given by

$$w = \begin{bmatrix} a+b & a-b \\ 1 & 1\end{bmatrix}. \tag{6}$$

with $a = \omega\cot(\omega\Delta t)$, and $b = \frac{|\csc(\omega\Delta t)|}{2\sqrt{2}\zeta}\sqrt{2-\zeta^2-\zeta^2\cos(2\omega\Delta t)}$. The eigenvalues are then

$$\begin{aligned}\lambda_1 &= 1-\exp(-\Delta t)\left(\frac{1}{4\omega^2\zeta^2} - \frac{\cos(2\omega\Delta t)}{4\omega^2} + \frac{|\sin(\omega\Delta t)|}{2\sqrt{2}\omega^2\zeta}\sqrt{2-\zeta^2-\zeta^2\cos(2\omega\Delta t)}\right) \\ \lambda_2 &= 1-\exp(-\Delta t)\left(\frac{1}{4\omega^2\zeta^2} - \frac{\cos(2\omega\Delta t)}{4\omega^2} - \frac{|\sin(\omega\Delta t)|}{2\sqrt{2}\omega^2\zeta}\sqrt{2-\zeta^2-\zeta^2\cos(2\omega\Delta t)}\right)\end{aligned} \tag{7}$$

The transformation matrix, $A_\beta$, will now depend on the parameters of the stimulus. Hence, we now refer to this matrix as $A_\beta(\zeta, \Delta t)$, illustrating its functional dependence on those parameters.

Some general intuition can be gained from the form of the above expressions. The eigenvalue gap, $\lambda_1 - \lambda_2$ is proportional to $\frac{\exp(-\Delta t)\|2\sin(\omega\Delta t)\|}{\zeta}$. Intuitively, the eigenvalue gap corresponds the relative importance of the two coding dimensions given by the eigenvectors of the regression matrix. The larger the eigenvalue gap, the more emphasis there is the eigenvector with lower eigenvalue for efficient predictive coding. If the eigenvalue gap is small, there is little benefit for prediction in measuring one dimension over the other. The nature of the dimensions to be measured depends on the direction of the eigenvectors of the regression matrix. This suggests that the eigenvalue gap grows for small $\Delta t$, then shrinks for large $\Delta t$. Additionally, in the small $\Delta t$ limit, the eigenvectors align strongly along the position and velocity axes, with the eigenvector corresponding to the smaller eigenvalue being along the position axis. Hence, for predictions with small $\Delta t$, the representation variable must encode a lot of information

about the position dimension. For longer timescale predictions, both eigenvectors contribute to large levels of compression, suggesting that the encoding scheme should feature a mix of both position and velocity. This is presented in Fig 4 (main text).

We also compute the total amount of predictive information available in this stimulus. This is given by

$$I(X_t; X_{t+\Delta t}) = \frac{1}{2}\log(|\Sigma_{X_t}|) - \frac{1}{2}\log(|\Sigma_{X_t|X_{t+\Delta t}}|). \tag{8}$$

Simplifying this expression yields

$$I(X_t; X_{t+\Delta t}) = \Delta t - \frac{1}{2}\log\Bigg( \exp(2\Delta t) + \cos^4(\omega\Delta t) - \sin^4(\omega\Delta t) \\ -2\exp(\Delta t)\left(\cos^2(\omega\Delta t) + \frac{1+\zeta^2}{1-\zeta^2}\sin^2(\omega\Delta t)\right) + 2\sin^2(\omega\Delta t)\Bigg) \tag{9}$$

We can see for very large $\Delta t$, this expression becomes

$$I(X_t; X_{t+\Delta t}) \sim \Delta t - \frac{1}{2}\log\left(\exp(2\Delta t) - 2\exp(\Delta t)\right). \tag{10}$$

For small $\Delta t$, we note there are two conditions: $|\Sigma_{X_t|X_{t+\Delta t}}| < k$ and $|\Sigma_{X_t|X_{t+\Delta t}}| > k$, where $k$ corresponds to width of the distribution. If the width of the Gaussian is below $k$, we treat this as being effectively deterministic. In this case,

$$I(X_t; X_{t+\Delta t}) \propto \frac{1}{2}\log(|\Sigma_{X_t}|) \tag{11}$$

where there are some constants that set the units of the information and the reference point. For widths larger than $k$, the expression becomes:

$$I(X_t; X_{t+\Delta t}) \propto \exp(-\Delta t) \tag{12}$$

# C Comparing the information bottleneck Method to Different Encoding Schemes

We compare the encoding scheme discovered by the information bottleneck to alternate encoding schemes. We accomplish this by computing the optimal transformation for a particular parameter set for some value of $\beta$, $A_\beta(\zeta, \Delta u)$. We then determine the conditional covariance matrix, $\Sigma_{X_t|\tilde{X}}$. We generate data from this distribution and apply a two-dimensional unitary rotation. We then compute the covariance of the rotated data. This gives us a suboptimal encoding scheme, as represented in Figure 5b in yellow. We note that this representation contains the same amount of mutual information with the past as the optimal representation variable, though the dimensions the suboptimal encoding scheme emphasizes are very different. Evolving the rotated data forward in time and then taking the covariance of the resulting coordinate set gives us $\Sigma_{X_{t+\Delta t}|\tilde{X}}$, as plotted in Fig 5B in purple. We clearly see that encoding the past with the suboptimal representation reduces predictive information, as the predictions of the future are much more uncertain.

# D Comparing the information bottleneck method to Kalman filters

An alternative approach to predictive coding is Kalman filtering. Kalman filter-based approaches fuses predictions of a system's coordinates at a given time and historical

observations of the system's coordinates to achieve increased certainty about the future coordinates of the system [3]. However, despite the high-level similarity between Kalman filtering and the information bottleneck method, there are key differences making each technique unique. To show this difference, we present the mathematical structure of a Kalman filter:

$$\begin{aligned} X^{(\text{naive})}(\Delta t) &= \mathcal{H}(\Delta t) X(0) + \xi(\Delta t) \\ \tilde{X}^{(\text{measured})}(\Delta t) &= \mathcal{O} X(\Delta t) + \chi(\Delta t) \\ X^{(\text{corrected})} &= X^{(\text{naive})}(\Delta t) + K_{\Delta t}(\tilde{X}^{(\text{measured})}(\Delta t) - \mathcal{O} X^{(\text{naive})}(\Delta t)). \end{aligned} \tag{13}$$

Here, $\mathcal{O}$ represents a measurement map and $\mathcal{H}(\Delta t)$ represents a dynamical systems model. These features are given to the Kalman filter by the designer. $K_{\Delta t}$ is the filter, and is a function of the measurement map, the dynamical systems model, and the prior uncertainty in the coordinates of the system. The Kalman filter is applied iteratively on each success $\Delta t$.

The structure of the Kalman filter reveals two key differences. First, the Kalman filter focuses on the decoding aspect of predictive coding, and is used to improve estimates of a predicted future coordinate via the measurement map and the dynamical systems model. However, the information bottleneck method focuses on the encoding aspect of this problem and generates an optimal encoding scheme for the past. Decoding is not considered explicitly in the information bottleneck. Second, because of the iterative structure of the Kalman Filter, it can use information from an extended time window into the past, while the information bottleneck method can only use one time point of information for predictive coding. This results in Kalman-filtering based approaches using more information about the past than necessary, resulting in inefficient predictive coding. We illustrate this in S1 Fig.

# E An approach to encoding when the parameters of the stimulus are evolving

We examine prediction in the SDDHO when the underlying parameters governing the trajectory are evolving faster than adaptation timescales. While there are many possible strategies for prediction in this regime, we consider a strategy where the system picks a representation that provides a maximal amount of information across a large family of stimulus parameters. We chose this strategy because it enables us to analyze the transferability of representations from one parameter set against another. In other words, we can understand how robust representations learned for particular stimulus parameters are.

We first determine the predictive information extracted by an efficient coder for a particular representation level, $I_{\text{past}}$ for a particular stimulus with parameters $(\zeta, \Delta t)$, $I^{\text{future}}_{\text{optimal}}((\zeta, \Delta t), I_{\text{past}})$. This predictive mapping is achieved by having a mapping, $\mathcal{P}(\tilde{X}|X_t)$. We apply this mapping to a new stimulus with different parameters $(\zeta, \Delta t)$ to determine the amount of predictive information extracted by this mapping on a different stimulus with parameters $(\zeta', \Delta t')$. We call this predictive information $I^{\text{future}}_{\text{transfer}}((\zeta, \Delta t), I_{\text{past}} \to (\zeta', \Delta t'))$.

We quantify the quality of these transferred representations in comparison with $I^{\text{future}}_{\text{optimal}}((\zeta', \Delta t'), I_{\text{past}})$ as

$$Q^{\text{transfer}}((\zeta,\Delta t)) = \frac{\int_{\Delta t_{\min}}^{\Delta t_{\max}} \int_{\zeta_{\min}}^{\zeta_{\max}} I_{\text{transfer}}^{\text{future}}((\zeta,\Delta t), I_{\text{past}} \to (\zeta',\Delta t'))d\zeta' d\Delta t'}{\int_{\Delta t_{\min}}^{\Delta t_{\max}} \int_{\zeta_{\min}}^{\zeta_{\max}} I_{\text{optimal}}^{\text{future}}((\zeta',\Delta t'), I_{\text{past}} d\zeta' d\Delta t'} \tag{14}$$

The resulting value is the performance of the mapping against a range of stimuli. In Figure 6, we analyzed the performance of mappings learned on $\frac{1}{3} < \zeta < 3$, $0.1 < t < 10$, on stimuli with parameters $\frac{1}{3} < \zeta' < 3$, $1 < t' < 10$. This choice of range is somewhat arbitrary, but it is large enough to see the asymptotic behavior in $\Delta t$, $\zeta$.

# F History Dependent Harmonic Oscillators

We extend the results on the Stochastically Driven Damped Harmonic Oscillator to history-dependent stimuli by modifying the original equations of motion to have a history dependent term using the Generalized Langevin Equation

$$\begin{aligned}\frac{dv}{dt} &= -\int_0^t \frac{\gamma v}{|t-t'|^\alpha} dt' - \omega_0^2 x + \xi(t) \\ \frac{dx}{dt} &= v\end{aligned} \tag{15}$$

where $-\frac{\gamma}{|t-t'|^\alpha}$ governs how the history impacts the velocity-position evolution. In the main text, we take $\gamma = 1$, $\omega = 1$, and $\alpha = 5/4$. To compute the autocorrelation functions, we compute the Laplace transform of each autocorrelation function and numerically invert the Laplace transform to estimate the value

$$\begin{aligned}\mathcal{L}[\langle v(t)v(0)\rangle] &= \frac{s}{s^2+\gamma s^\alpha+\omega^2} \\ \mathcal{L}[\langle v(t)x(0)\rangle] &= -\frac{1}{s^2+\gamma s^\alpha+\omega^2} \\ \mathcal{L}[\langle x(t)v(0)\rangle] &= -\mathcal{L}[\langle v(t)x(0)\rangle] \\ \mathcal{L}[\langle x(t)x(0)\rangle] &= \frac{1}{\omega^2 s} - \frac{1}{s^2+\gamma s^\alpha+\omega^2}.\end{aligned} \tag{16}$$

To expand our past and future variables to include multiple time points, we extend the past variable to be observations between $t - t_0$ and $t$ and the future variable to be $t + \Delta t$ to $t + \Delta t + t_0$. The size of the window is set by $t_0$. We discretize each window with a spacing of $dt = 1$ and compute correlation functions along the discrete points of time, yielding the full covariance matrices. Ideally, we would make the discretization interval arbitrarily small, $dt \to 0$. However, this introduces numerical issues, as the determinant of $\Sigma_{t-t_0:t}$ approaches 0. As such, we make $dt$ as small as possible without causing this numerical issue. We explore a few values of $dt$ in S2 Fig to determine the effect on the information curve. While small $dt$ confers more information, there are diminishing returns and we asymptotically approach the correct values. After this, the recipe is as outlined in S1 Text.

# References

1. Nørrelykke SF, Flyvbjerg H. Harmonic oscillator in heat bath: Exact simulation of time-lapse-recorded data and exact analytical benchmark statistics. Phys Rev E. 2011;83:041103. doi:10.1103/PhysRevE.83.041103.

2. Sandev T, Metzler R, Tomovski v. Correlation functions for the fractional generalized Langevin equation in the presence of internal and external noise. Journal of Mathematical Physics. 2014;55(2):023301. doi:10.1063/1.4863478.

3. Kalman RE. A New Approach to Linear Filtering and Prediction Problems. Transactions of the ASME–Journal of Basic Engineering. 1960;82(Series D):35–45.

4. Laughlin SB. A Simple Coding Procedure Enhances a Neuron's Information Capacity. Zeitschrift für Naturforschung C. 1981;36:910 – 912.

5. Chechik G, Globerson A, Tishby N, Weiss Y. Information Bottleneck for Gaussian Variables. In: Thrun S, Saul LK, Schölkopf B, editors. Advances in Neural Information Processing Systems 16. MIT Press; 2004. p. 1213–1220. Available from: `http://papers.nips.cc/paper/2457-information-bottleneck-for-gaussian-variables.pdf`.

6. Srinivasan MV, Laughlin SB, Dubs A, Horridge GA. Predictive coding: a fresh view of inhibition in the retina. Proceedings of the Royal Society of London Series B Biological Sciences. 1982;216(1205):427–459. doi:10.1098/rspb.1982.0085.
