# Supplementary material for: Optimal prediction with resource constraints using the information bottleneck
Source: PLoS Comput Biol. 2021 Mar 8;17(3):e1008743. doi: 10.1371/journal.pcbi.1008743 (PMC7971903; doi:10.1371/journal.pcbi.1008743)
Supplement: S3 Text — We provide some detail about the parameters in our simulation for Wright-Fisher dynamics and a short derivation for the maximum amount of encoded information for a given m. (PDF) [file pcbi.1008743.s003.pdf]

S3 Text: Wright Fisher Dynamics

February 3, 2021

Wright-Fisher dynamics are used in population genetics to describe the evolution of a population of fixed size over generations. Here, we consider the diffusion approximation to the Wright-Fisher model with continuous time, given by Eq.18. We numerically integrate Eq.18 using a time step of $dt = 0.001$ and use 10000 data points starting from a given initial allele frequencies to estimate the joint distribution, $P(X_{t+\Delta t}, X_t)$. We discretize allele frequency space with $N+1$ bins. We compute the maximum available predictive information for different values of the parameters (Figure 10) using

$$I(X_t; X_{t+\Delta t}) = -\sum_{X_t} \mathcal{P}(X_t) \log(\mathcal{P}(X_t)) - \sum_{X_{t+\Delta t}} \mathcal{P}(X_{t+\Delta t}) \log(\mathcal{P}(X_{t+\Delta t})) + \sum_{X_t, X_{t+\Delta t}} \mathcal{P}(X_t, X_{t+\Delta t}) \log(\mathcal{P}(X_{t+\Delta t}, X_t)). \quad (1)$$

A simple estimate for $I(X_t; \tilde{X})$ can be obtained by considering the case where each individual memory reflects a distinct cluster of allele frequencies. In the optimal encoding case, each memory encodes an equal amount of probability weight on the input variable [1, 2]. The upper bound on the information the representation variable has about the past state is $I(X_t; \tilde{X}) = \log(m)$.

References

1. Laughlin SB. A Simple Coding Procedure Enhances a Neuron's Information Capacity. Zeitschrift für Naturforschung C. 1981;36:910 – 912.

2. Srinivasan MV, Laughlin SB, Dubs A, Horridge GA. Predictive coding: a fresh view of inhibition in the retina. Proceedings of the Royal Society of London Series B Biological Sciences. 1982;216(1205):427–459. doi:10.1098/rspb.1982.0085.
