# Supplementary figures and images for: Optimal prediction with resource constraints using the information bottleneck
Source: PLoS Comput Biol. 2021 Mar 8;17(3):e1008743. doi: 10.1371/journal.pcbi.1008743 (PMC7971903; doi:10.1371/journal.pcbi.1008743)

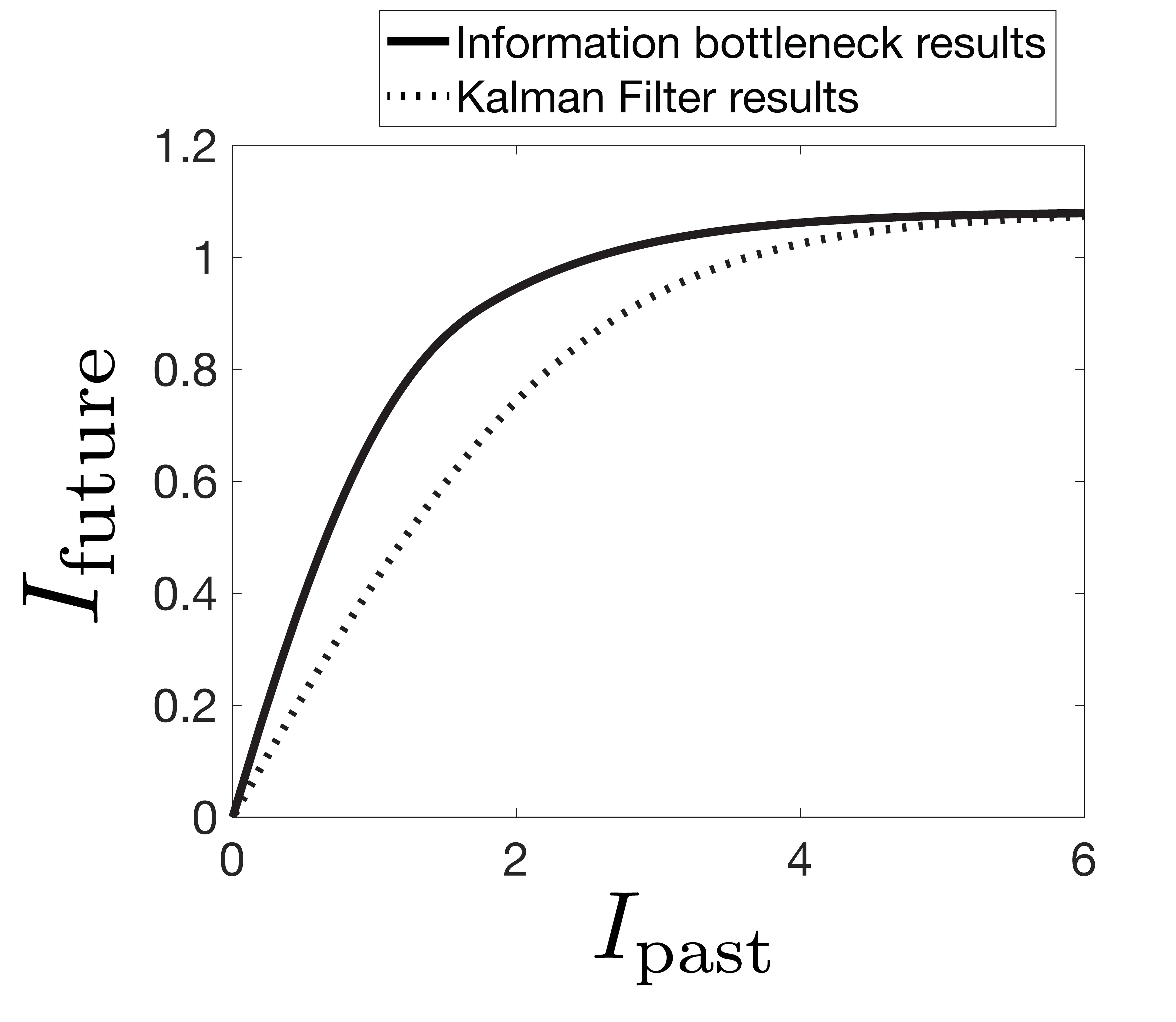

Supplement: S1 Fig — We compare the amount of information conferred about the future for a given encoding level and find that Kalman Filter-based approaches do not maximize the amount of predictive information conferred, suggesting they are not efficient predictive coding schemes. (TIF) [file pcbi.1008743.s004.tif]

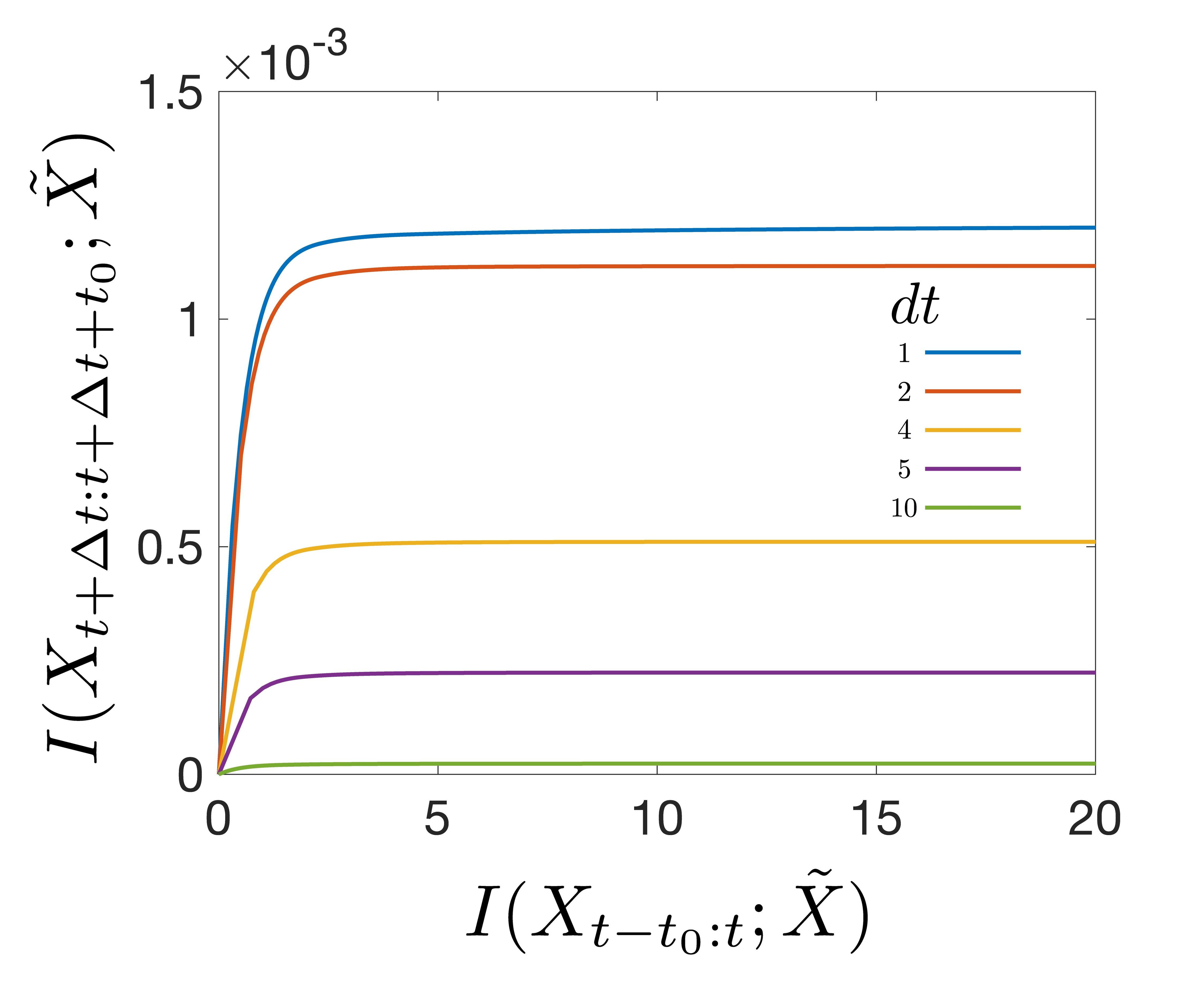

Supplement: S2 Fig — We note that there are diminishing returns for increasingly small dt. However, we cannot make dt arbitrarily small, as this introduces numerical errors. (TIF) [file pcbi.1008743.s005.tif]

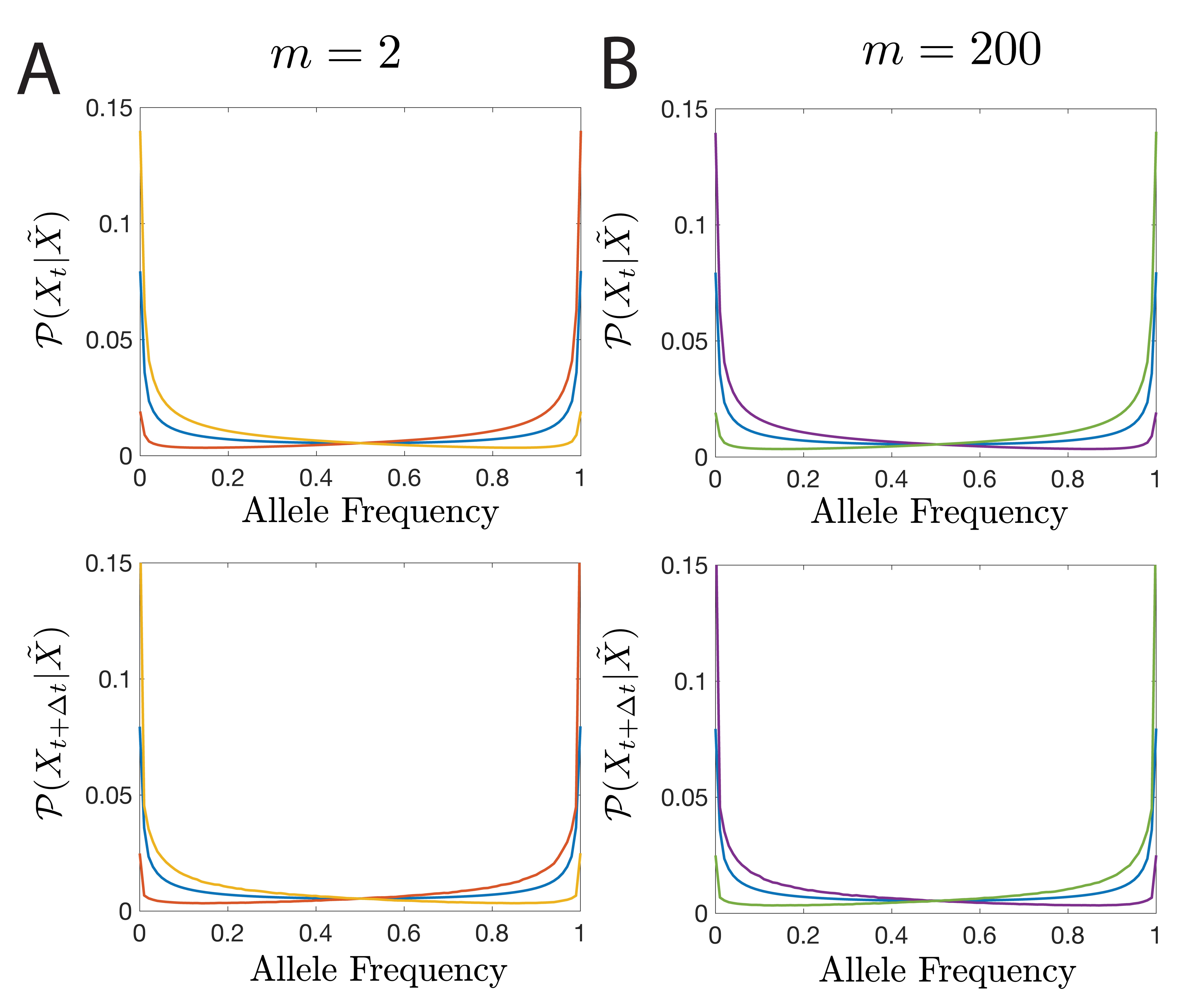

Supplement: S3 Fig — Many representations are degenerate in the m = 200 in this limit. The encoding schemes for m = 2 versus m = 200 are nearly identical for this small I(Xt;X˜) limit. (TIF) [file pcbi.1008743.s006.tif]

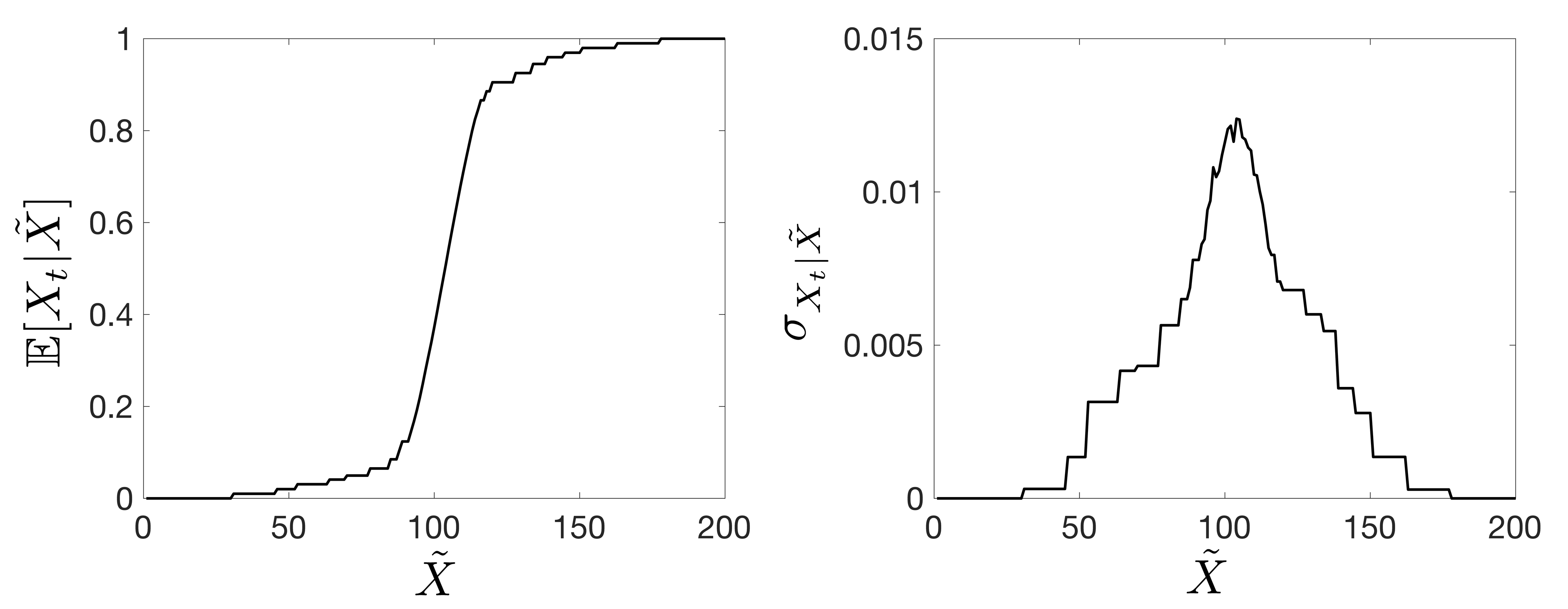

Supplement: S4 Fig — The standard deviation is not constant: it is smaller where the prior probability of Xt is large. (TIF) [file pcbi.1008743.s007.tif]
